# Supplementary material for: Auditing the readiness of healthcare facilities for referral and management of pre-eclampsia cases in Zanzibar- a study protocol
Source: PLoS One. 2023 Jun 2;18(6):e0286498. doi: 10.1371/journal.pone.0286498 (PMC10237472; doi:10.1371/journal.pone.0286498)
Supplement: S2 File — (DOCX) [file pone.0286498.s002.docx]

**A check list for assessing availability of medication, equipment and tests related to management of pre-eclampsia**

**Characteristics of health facilities**

1. Name of HF
2. Level of HF ( )
3. PHCU ( )
4. PHCU+ ( )
5. PHCC ( )
6. DISTRICT HOSPITAL
7. REGIONAL HOSPITAL ( )
8. TERTIARY HOSPITAL ( )

3. Human resource ……………………………

- 1. Specialist……………………….
  2. Medical doctor…………………
  3. Assistant medical officer……….
  4. Clinical Officer…………………
  5. Nurses…………………………..

**Availability of the equipment for referral and management of Preeclampsia/Eclampsia**

| SN | ITEAM | AVAILABLE | NOT AVAILABLE |
| --- | --- | --- | --- |
| 1 | Guidelines/protocols/poster for the management of pre-eclampsia |  |  |
| 2 | Working BP machine |  |  |
| 3 | Working Stethoscope |  |  |
| 4 | Working Fetoscope |  |  |
| 5 | Working Ambu bag |  |  |
| 6 | Working Oxygen  cylinders/ Concentrators. |  |  |
| 7 | Working Patellar hammer |  |  |
| 8 | Urinary catheters(Foley catheter) |  |  |
| 9 | Urinary bags |  |  |
| 10 | Drip stands |  |  |
| 11 | Syringes (10cc/20cc) |  |  |
| 12 | Strips to detect proteinuria |  |  |
| 13 | Cannula |  |  |
| 14 | Intravenous giving set |  |  |

**Availability of drugs for the management of pre-eclampsia and eclampsia**

|  | Drug | Available | Not Available |
| --- | --- | --- | --- |
| 21 | Magnesium sulfate |  |  |
| 22 | Diazepam |  |  |
| 23 | Phenytoin |  |  |
| 24 | Calcium gluconate 10% |  |  |
| 25 | Hydralazine |  |  |
| 26 | Dexamethasone |  |  |
| 27 | Aldomet/ Methyldopa |  |  |
| 28 | Nifedipine |  |  |
| 29 | Misoprostol |  |  |
| 30 | Oxytocin |  |  |

**Availability of diagnostic test**

| **SN** | Diagnostic | Available | Not available |
| --- | --- | --- | --- |
|  | Urine protein test |  |  |
|  | Renal function test |  |  |
|  | Liver function test |  |  |
