# Supplementary material for: Auditing the readiness of healthcare facilities for referral and management of pre-eclampsia cases in Zanzibar- a study protocol
Source: PLoS One. 2023 Jun 2;18(6):e0286498. doi: 10.1371/journal.pone.0286498 (PMC10237472; doi:10.1371/journal.pone.0286498)
Supplement: S3 File — (DOCX) [file pone.0286498.s003.docx]

A checklist for the management of pre-eclampsia

1. Name of HF…………………………

2. Level of HF ( )

a) PHCU ( )

b) PHCU+ ( )

c) PHCC ( )

d) DISTRICT HOSPITAL

e) REGIONAL HOSPITAL ( )

f) TERTIARY HOSPITAL ( )

3. Number of health care providers ………….

a) Specialist……………………….

b) Medical doctor…………………

c) Assistant medical officer……….

d) Clinical Officer…………………

e) Nurses…………………………..

| SN | ITEM | YES | NO | Missing data |
| --- | --- | --- | --- | --- |
|  | **MILD PRE ECLAMPSIA diastolic ≥90mmhg <110mmhg with proteinuria +1 or +2** |  |  |  |
|  | MANAGEMENT |  |  |  |
| 1 | BP monitoring every 4hrs |  |  |  |
| 2 | Fetal heart rate recorded 4HRLY |  |  |  |
| 3 | 24 hours of urine for protein is checked |  |  |  |
| 4 | If pregnancy < 37-week corticosteroids started |  |  |  |
| 5 | The patient is on methyldopa 500mg 8hourly |  |  |  |
|  | **SEVERE when BP ≥ 160/110 mmHg (especially diastolic ≥110 mmHg), Proteinuria ≥+++or ≥ 1g/24h, presenting with severe headache, epigastric pain, blurring of vision +/** |  |  |  |
| 6 | Hydralazine started (if not available check for number 7) |  |  |  |
| 7 | Nifedipine: 10 mg (PO) short-acting started |  |  |  |
| 8 | If hypertension is noncompliant to hydralazine. Labetalol started (10–20mg intravenously bolus repeated each 10–20 minutes, with doubling doses not exceeding 80 mg in any single dose for maximum total cumulative dose) |  |  |  |
| 9 | The patient kept on Nifedipine: 20 mg (PO) 8 hourly |  |  |  |
| 10 | The loading dose of magnesium sulfate started |  |  |  |
| 11 | The patient received or on the magnesium maintenance dose |  |  |  |
| 12 | 24hrs urine for protein |  |  |  |
| 13 | BP monitoring |  |  |  |
| 14 | Fetal heart rate monitoring |  |  |  |
| 15 | Urine output, maternal reflexes, respiratory rate are evaluated |  |  |  |
| 16 | The first dose of steroids are given for fetal lung maturity if the fetus is between 24-34 weeks gestational age |  |  |  |
| 17 | There is a well-defined written protocol for the management of eclampsia available and in the facility |  |  |  |
