# Supplementary material for: Auditing the readiness of healthcare facilities for referral and management of pre-eclampsia cases in Zanzibar- a study protocol
Source: PLoS One. 2023 Jun 2;18(6):e0286498. doi: 10.1371/journal.pone.0286498 (PMC10237472; doi:10.1371/journal.pone.0286498)
Supplement: S4 File — (DOCX) [file pone.0286498.s004.docx]

A checklist for assessing the Referral Standards within lower level health care facilities

| **Variable** | **Available** | **Not available** |
| --- | --- | --- |
| Availability of guideline for referral |  |  |
| A clear referral pathway established |  |  |
| A clear criteria for patient with pre-eclampsia to be refer |  |  |
| Patient with pre-eclampsia receive pre referral treatment according to guideline |  |  |
| Referred patient with pre-eclampsia referred with referral note stating condition/ treatment given |  |  |
| Referred patient with pre-eclampsia accompanied with healthcare provider |  |  |
| Availability of transport to refer patient with signs of pre-eclampsia |  |  |
