# Supplementary material for: Auditing the readiness of healthcare facilities for referral and management of pre-eclampsia cases in Zanzibar- a study protocol
Source: PLoS One. 2023 Jun 2;18(6):e0286498. doi: 10.1371/journal.pone.0286498 (PMC10237472; doi:10.1371/journal.pone.0286498)
Supplement: Attachment — Submitted filename: PONE-D-21-13246_R2_reviewer_ Aug 31.pdf [file pone.0286498.s008.pdf]

PLOS ONE

Auditing the Readiness of Healthcare Facilities in Antenatal Care Referral and Management of Pre-Eclampsia in Zanzibar- A Protocol for Cross Sectional Study

--Manuscript Draft--

**Manuscript Number:**	PONE-D-21-13246R2
**Article Type:**	Study Protocol
**Full Title:**	Auditing the Readiness of Healthcare Facilities in Antenatal Care Referral and Management of Pre-Eclampsia in Zanzibar- A Protocol for Cross Sectional Study
**Short Title:**	Pre-eclampsia referral, management and healthcare facility readiness
**Corresponding Author:**	Salma Ali Rashid, MSc<br>University of Dodoma College of Health and Allied Sciences: The University of Dodoma College of Health Sciences<br>Dodma, Tanzania TANZANIA, UNITED REPUBLIC OF
**Keywords:**	Pre-eclampsia; eclampsia; healthcare facilities. Antenatal Care, referral, management, Zanzibar
**Abstract:**	Hypertensive disorders of pregnancy reported as the second leading root of maternal morbidity and mortality in Zanzibar. Evidences show that majority of pregnant women in Zanzibar are referred late from antenatal clinics and most end up with eclampsia. The objective of this study is to determine the prevalence of pre-eclampsia, and to assess the readiness of health care facilities in relation to pre-eclampsia referral and management in Zanzibar.<br>This is a descriptive cross-sectional study that will involve a total of 54 healthcare facilities, 176 health care providers and 138 pregnant women with gestation age of 20 weeks and above. Health care facilities will be selected by systematic random sampling, and simple random sampling will be used to select health care providers and pregnant women. Protein in urine test and measurement of blood pressure will be conducted for the diagnosis of pre-eclampsia. Healthcare facilities will be assessed if they meet the requirement for referral and for the availability of equipment and supplies, medications and lab-tests related to the management of pre-eclampsia using a pre-prepared checklist. Questionnaire will be used to determine the proportion of healthcare providers with knowledge and skill on the management of pre-eclampsia within each facility, and to obtain background characteristics information from pregnant women, while patients case files in each facility will be reviewed to assess the management of pre-eclampsia using a pre-prepared checklist. Data will be analysis using SPSS software version 25. Descriptive statistics will be used to describe the frequency distribution of the study variables and results will be presented in frequency and percentage. Chi square test will be used to describe the relationship between variables and the p-value of < 0.05 will be regarded as statistically significant difference.
**Order of Authors:**	Salma Ali Rashid, MSc
	Saada Ali Seif, PhD
**Response to Reviewers:**	1. Introduction section<br>i. Please focus this part on the facts according to title of you study. Don’t repeat the WHO guidelines and available treatment options. Explain what the deficiencies or berries in implementations of these guidelines in your area or hospitals are and how you can rectify them.<br><br>Response:This section has been re written line 69-170<br><br>ii. Line 90-92, please insert the year to which this data belongs, page 3.<br><br>Response:The year is inserted (line 108-110)<br><br>iii. Please add reference to the statement made in line 149-150, page 5.<br><br>Response:The reference is added (line 164)

Powered by Editorial Manager® and ProduXion Manager® from Aries Systems Corporation

	2. Study area section<br>i. Line 208-215, page 8; This statement is repetition of last paragraph of introduction part. So, it can be omitted.<br><br>Response:The paragraph is omitted<br><br>3. Variable definition sections<br>i. Please don't repeat the definition of pre-eclampsia (line 262-264, and line 272-276 at page 10. This section needs to be worked on and rephrased.<br><br>Response:The section has been omitted but the important information is included in variables measurement section (line 287-305)<br><br>ii. Authors still have not made it clear how information will be collected in tool, whether it will be a smartphone or computer-based form or a printed questionnaire will be kept at the hospitals<br><br>Response:This information is included in line 346-349<br><br>iii. Similarly, there will be different categories of health care workers like medical graduates, nursing staff and other paramedical staff at each hospital. Authors should explain how they will prevent bias while collecting information from them.<br><br>Response:For healthcare providers, self-administered questionnaire will be used. The same standardized questionnaire will be used to all categories and results will be presented for each healthcare category. This information is added in line 316-317.<br><br>4. Independent variables<br>i. This section as whole need to be rewritten, sentences is framed very badly. e.g line 298. Page 11; The tool will be adopted from [29] and will be little modified to suit the current study.<br><br>Response:The section is re-written line 311-336<br><br>Other comments<br>i. Authors are requested to work on English language in manuscript. Although, they have improved a lot, but much more needs to be done.<br><br>Response: The manuscript was sent to English editor<br><br>Please use uniform method of writing pre-eclampsi/eclampsia instead of writing PE/E or full form randomly. Similarly use blood pressure instead of writing Blood Pressure.<br><br>Response: This has been corrected in the whole document<br><br>ii. Authors have not improved reference section. Some references are incomplete. e.g. Reference number at 4, 6, 7, 9, 18, 21, and 25. Please rewrite them.<br>Response:References are now corrected (number 4,12,13,15,19,23). After editing the text one reference was dropped
**Additional Information:**	
**Question**	**Response**
**Financial Disclosure**<br><br>Enter a financial disclosure statement that describes the sources of funding for the work included in this submission. Review the submission guidelines for detailed requirements. View published research articles from *PLOS ONE* for specific	The authors received no specific funding for this work

examples.<br><br>This statement is required for submission and **will appear in the published article** if the submission is accepted. Please make sure it is accurate.<br><br>**Unfunded studies**<br>Enter: *The author(s) received no specific funding for this work.*<br><br>**Funded studies**<br>Enter a statement with the following details:<br>• Initials of the authors who received each award<br>• Grant numbers awarded to each author<br>• The full name of each funder<br>• URL of each funder website<br>• Have or will the sponsors or funders play any role in the study design, data collection (when applicable) and analysis, decision to publish, or preparation of the manuscript?<br>• **NO** - Include this sentence at the end of your statement: *The funders had and will not have a role in study design, data collection and analysis, decision to publish, or preparation of the manuscript.*<br>• **YES** - Specify the role(s) played.<br><br>* typeset	
**Competing Interests**<br><br>Use the instructions below to enter a competing interest statement for this submission. On behalf of all authors, disclose any competing interests that could be perceived to bias this work—acknowledging all financial support and any other relevant financial or non-financial competing interests.<br><br>This statement is required for submission and **will appear in the published article** if the submission is accepted. Please make sure it is accurate and that any funding sources listed in your Funding Information later in the submission form are also declared in your Financial Disclosure statement.	The authors have declared that no competing interests exist

View published research articles from *PLOS ONE* for specific examples.<br><br>**NO authors have competing interests**<br><br>Enter: *The authors have declared that no competing interests exist.*<br><br>**Authors with competing interests**<br><br>Enter competing interest details beginning with this statement:<br><br>*I have read the journal's policy and the authors of this manuscript have the following competing interests: [insert competing interests here]*<br><br>* typeset	
**Ethics Statement**<br><br>Enter an ethics statement for this submission. This statement is required if the study involved:<br><br>• Human participants<br>• Human specimens or tissue<br>• Vertebrate animals or cephalopods<br>• Vertebrate embryos or tissues<br>• Field research<br><br>Write "N/A" if the submission does not require an ethics statement.<br><br>General guidance is provided below. Consult the submission guidelines for detailed instructions. **Make sure that all information entered here is included in the Methods section of the manuscript.**	The ethical permission was obtained from University of Dodoma Research ethical committee (UDOM-REC) with reference number No.MA.84/261/02/218<br>and Zanzibar Health Research Institute with reference NO. ZAHREC/04/ST/JAN/2021/02<br>A written consent will be obtained.

Powered by Editorial Manager® and ProduXion Manager® from Aries Systems Corporation

**Format for specific study types**<br><br>**Human Subject Research (involving human participants and/or tissue)**<br>• Give the name of the institutional review board or ethics committee that approved the study<br>• Include the approval number and/or a statement indicating approval of this research<br>• Indicate the form of consent obtained (written/oral) or the reason that consent was not obtained (e.g. the data were analyzed anonymously)<br><br>**Animal Research (involving vertebrate animals, embryos or tissues)**<br>• Provide the name of the Institutional Animal Care and Use Committee (IACUC) or other relevant ethics board that reviewed the study protocol, and indicate whether they approved this research or granted a formal waiver of ethical approval<br>• Include an approval number if one was obtained<br>• If the study involved *non-human primates*, add *additional details* about animal welfare and steps taken to ameliorate suffering<br>• If anesthesia, euthanasia, or any kind of animal sacrifice is part of the study, include briefly which substances and/or methods were applied<br><br>**Field Research**<br><br>Include the following details if this study involves the collection of plant, animal, or other materials from a natural setting:<br>• Field permit number<br>• Name of the institution or relevant body that granted permission	
**Data Availability**<br><br>Authors are required to make the data underlying their research findings fully available, without restriction.<br><br>If the manuscript reports pilot or preliminary results, the data underlying those results must be made available at the time of publication. A Data Availability	N/A - the protocol does not report results

Statement describing where the data can be found is required at submission. Your answers to this question constitute the Data Availability Statement and will be published in the article, if accepted. When legal or ethical requirements prohibit public data sharing, authors must indicate how researchers may obtain access to the data. See the PLOS Data Policy and FAQ for detailed information.<br><br>**Important:** Stating 'data available on request from the author' is not sufficient. If your data are only available upon request, select 'No' for the first question and explain your situation in the text box.<br><br>For protocols without pilot or preliminary data, authors are strongly encouraged to state how they plan to share research data from their study when it is completed or published.<br><br>Do the authors confirm that EITHER the protocol does not report results OR all data underlying the preliminary results will be made fully available without restriction?	
**Describe where the data may be found in full sentences. If you are copying our sample text, replace any instances of XXX with the appropriate details.**<br><br>• If **no pilot data are reported** in the manuscript, please enter the following: *No datasets were generated or analysed during the current study. All relevant data from this study will be made available upon study completion.*<br>• If the manuscript reports pilot data **held in a public repository**, include URLs, accession numbers or DOIs. If this information will only be available after acceptance, indicate this by ticking the box below. For example: *All XXX files are available from the XXX database*	Deidentified research data will be made publicly available when the study is completed and published

(accession number(s) XXX, XXX.).<br>• If the manuscript reports pilot data contained **within the manuscript and/or Supporting Information files**, enter the following:<br>*All relevant data are within the manuscript and its Supporting Information files.*<br>• If neither of these applies but you are able to provide **details of access elsewhere**, with or without limitations, please do so. For example:<br><br>*Data cannot be shared publicly because of [XXX]. Data are available from the XXX Institutional Data Access / Ethics Committee (contact via XXX) for researchers who meet the criteria for access to confidential data.*<br><br>*The data underlying the results presented in the study are available from (include the name of the third party and contact information or URL).*<br>• This text is appropriate if the data are owned by a third party and authors do not have permission to share the data.<br><br>For study protocols with data management or sharing plans, authors are encouraged to briefly describe in their Data Availability Statement how data they generate will be made accessible when the study is completed. For example:<br>• Deidentified research data will be made publicly available when the study is completed and published.<br><br>* typeset	
Additional data availability information:	

Powered by Editorial Manager® and ProduXion Manager® from Aries Systems Corporation

Manuscript Click here to access/download;Manuscript;protocol clean third.docx

Auditing the Readiness of Healthcare Facilities in Antenatal Care Referral and Management of Pre-Eclampsia in Zanzibar- A Protocol for Cross Sectional Study

Salma A. Rashid[1¶*], Saada A. Seif[2¶]

[1] Department of Clinical Nursing, The University of Dodoma, Dodoma, Tanzania

[2]Department of Nursing Management and Education, The University of Dodoma, Dodoma, Tanzania

Funding: No fund received for this study

Competing interest: There is no competing interest to declare

Data availability: Deidentified research data will be made publicly available when the study is completed and published

[*]Corresponding author
Email: sallyrasheed.sr@gmail.com

[¶]These authors contributed equally to this work

Abstract

Hypertensive disorders of pregnancy reported as the second leading root of maternal morbidity and mortality in Zanzibar. Evidences show that majority of pregnant women in Zanzibar are referred late from antenatal clinics and most end up with eclampsia. The objective of this study is to determine the prevalence of pre-eclampsia, and to assess the readiness of health care facilities in relation to pre-eclampsia referral and management in Zanzibar.

This i[illegible] a descriptive cross-sectional study that will involve a total of 54 healthcare facilities, 176 health care providers and 138 pregnant women with gestation age of 20 weeks and above. Health care facilities will be selected by systematic random sampling, and simple random sampling will be used to select health care providers and pregnant women. Protein in urine test and measurement of blood pressure will be conducted for the diagnosis of pre-eclampsia. Healthcare facilities will be assessed if they meet the requirement for referral and for the availability of equipment and supplies, medications and lab-tests related to the management of pre-eclampsia using a pre-prepared checklist. Questionnaire will be used to determine the proportion of healthcare providers with knowledge and skill on the management of pre-eclampsia within each facility, and to obtain background characteristics information from pregnant women, while patients case files in each facility will be reviewed to assess the management of pre-eclampsia using a pre-prepared checklist. Data will be analysis using SPSS software version 25. Descriptive statistics will be used to describe the frequency distribution of the study variables and results will be presented in frequency and percentage. Chi square test will be used to describe the relationship between variables and the p-value of < 0.05 will be regarded as statistically significant difference.

Keywords:

Pre-eclampsia, eclampsia, healthcare facilities readiness, antenatal care referral, management of pre-eclampsia, Zanzibar.

Introduction

Pre-eclampsia is defined as a pregnancy complication characterized by high blood pressure of at least 140mmHg systolic and 90mmHg diastolic pressure measured on two occasions, 4 - 6 hours apart, accompanied by proteinuria of at least 300 mg per 24 hours, or at least +1mg on dipstick tested after 20 weeks of gestation age, and or with swelling of lower limbs, severe headache and or upper abdominal pain and or blurred vision or light sensitivity. While

eclampsia is the existence of a new-onset of grand mal-seizure in women with pre-eclampsia [1]. The pathogenesis of pre-eclampsia is not known, though it is associated with abnormal placentation at the beginning of pregnancy, while hypertension and proteinuria occurred in the second half of pregnancy [2]. The risk factors like obesity, chronic hypertension, diabetics, nulliparous, and twinning pregnancy have been shown to increase the likelihood of developing pre-eclampsia to pregnant women [3].

Pre-eclampsia is categorized as mild and severe, the mild one is diagnosed when blood pressure is greater than 140 systolic or 90 diastolic and 0.3mg of protein is collected in a 24-hour urine sample, or persistent of +1~~mg~~ protein measurement in urine dipstick. While severe one is diagnosed when the blood pressure is greater than 160/110 mmHg (especially diastolic ≥110 mmHg), proteinuria of 3+~~mg~~ or greater or 1~~mg~~ or greater in 24hours, with severe headache, epigastric pain, blurri[illegible]f vision and vomiting[4]. The prognosis from mild to severe can be fast and unpredictable if left untreated.

The prevalence of pre-eclampsia/eclampsia is reported to be much higher in developing countries which ranges from 1.8 to 16.7% [5], while in developed countries like United States of America, the pre-eclampsia prevalence ranges from 3-6% [6]. In East African countries, the prevalence of pre-eclampsia ranges from 6.1% in Kenya [7] to 12.2% in Ethiopia [8]. The recent study done in Tanzania showed the prevalence of severe pre-eclampsia is 4.2% [9] while in Zanzibar the prevalence of pre-eclampsia reported in the year 2011 was 9% [10]. Therefore, there is still need to determine the current prevalence of pre-eclampsia in the country to understand its trend.

Researches show that pre-eclampsia/eclampsia is among the primary cause of maternal and neonatal morbidity and mortality. It is reported to affect 2–8% of pregnancies globally, and it is associated with 10–15% of direct maternal deaths and up to 25% of stillbirths and newborn deaths in developing countries [11], [12]. Tanzania is reported to have 556 maternal deaths per 100,000 live birth [13], and eclampsia related complications accounts for 18.9% to maternal deaths [14]. In Zanzibar, the report of 20[illegible]howed that maternal mortality was 155 deaths per 100,000 live birth [15], while the report of 2017 showed that severe pre-eclampsia was found to contribute to 25.8% of maternal morbidity and 21.8% of maternal mortality [16].

Effective management of pre-eclampsia/eclampsia can be possible by following the WHO guideline of Antenatal Care (ANC) screening for early detection of signs of pre-eclampsia as

well as the following the guideline for pharmacological management and meet the specified requirement for referral. Poor adherence to these guidelines can result in poor management of pregnant women with signs of pre-eclampsia and thus may contributes to increasing of maternal morbidity and mortality related to pre-eclampsia/eclampsia.

Various factors that affect the management of pre-eclampsia/eclampsia have been identified, and these include unavailability of medication and supply and lack of healthcare providers with adequate knowledge and skills for the management of pre-eclampsia. All these may result in failure to early detect, diagnose and providing proper treatment of pre-eclampsia [17], [18]. Therefore, assessing these factors in each healthcare facility levels is very important step for enhancing good decision making for the distribution of medical supplies related to the management of pre-eclampsia/eclampsia at different healthcare facility levels.

The Ministry of Health Zanzibar adopted the WHO guideline and developed the ‘Management Protocols of Obstetric and Newborn Care’ which is to be uniformly followed at all healthcare facility levels. In addition, health care providers are provided with training on focused antenatal care which include the use of these guidelines. According to this protocol all pregnant women attending ANC must be screened for pre-eclampsia/eclampsia, and those showing signs of pre-eclampsia/eclampsia, must be appropriately managed, and those presented with severe pre-eclampsia at lower healthcare facility levels, must be referred to the next level immediately [19]. However, evidence showed that majority of pregnant women in Zanzibar are referred late and most ends up with eclampsia (25.8%)[16].

Hence the questions come of whether all levels of healthcare facilities in Zanzibar are prepared enough in terms of availability of the required equipment, medications, adherence to management and referral guidelines as well as the availability of healthcare providers with knowledge and skills for the management of pre-eclampsia/eclampsia. On this ground, this research therefore intends to assess the prevalence of pre-eclampsia and the healthcare facilities readiness in relation to ANC referral and management of pre-eclampsia in Zanzibar.

Study model: Donabedian model for measuring quality of care

This study will be guided by model for measuring quality care by Donabedian (Fig. 1) [20]. This model has three approaches to evaluation of the quality of care, which are structure, process, and outcome. The structure which is also known as input measure entails the characteristics of the healthcare facility such as staff to patient ratio and operating times and

hours of the services. Process measures reflect the way systems work to deliver the desired outcome, and the outcome measure reflects the impact on the patient and demonstrates the result of improvement and whether it has ultimately achieved the aim(s) set [20].

The current study will adopt all approaches as specified by the model as follows: The structure measures will be the availability of resources in the health care facilities like skilled and knowledgeable health care providers, availability of guidelines, availability of required tests, resources and equipment, on job training, availability of ambulance, and availability of medication. The process measure will be the adherence to the guideline on the treatment of pre-eclampsia/eclampsia and adherence of guidelines in making the referral to women with signs of severe pre-eclampsia. The outcome measure will be the number of emergency admissions due to severe pre-eclampsia and the number of patients with eclampsia.

Methods

Study objectives

The general objective of this study is to determine the proportion of pre-eclampsia and to assess the readiness of healthcare facilities in relation to pre-eclampsia antenatal care referral and its management in Zanzibar.

The Specific objectives are:

1. To determine the prevalence of pre-eclampsia among pregnant women attending ANC.
2. To assess the availability of equipment, medication and tests related to the management of pre-eclampsia among all healthcare facility levels.
3. To determine proportion of health care providers working in ANC with adequate knowledge on the management of pre-eclampsia
4. To determine proportion of health care providers working in ANC with adequate skills on the management of pre-eclampsia
5. To assess the management of pre-eclampsia in all healthcare facility levels
6. To assess the status of referral standards among healthcare facilities of lower levels

Study design

A descriptive cross-sectional design using a quantitative approach will be used.

Study area

This study will be conducted in Zanzibar which is united with and a part of Tanzania. The study setting will be healthcare facilities of all levels in Zanzibar. The healthcare delivery system in Zanzibar is organized into three levels which are the primary level, secondary and tertiary level. The primary level is the lowest level which includes Primary Health Care Unit (PHCU), Primary Health Care Unit Plus (PHCU +) and Primary Health Care Center or Cottage (PHCC). In total, there are 122 PHCU, 34 PHCU+, and 4 PHCC. Primary health care units provide primary health care services and all ANC services including screening of pre-eclampsia, while the PHCU+ provides additional services such as delivery, dental, laboratory, and pharmaceutical services while the PHCCs provide the same services as PHCU+ with the addition of inpatient and X-ray services.

The secondary level which is the middle level of health care delivery includes district hospitals and the regional hospitals. There are two district hospitals and one regional hospital. District

hospitals provide second-line referral services including basic surgery, and regional hospitals provide referral points for all district hospitals. The highest level of health care delivery is the tertiary level or called the referral hospital. There is only one referral hospital in Zanzibar which is Mnazi Mmoja National Hospital which provides referral services from the district and regional hospitals.

Study Population

This study will involve three types of study population[illegible]

1. All pregnant women attending ANC with gestation age of 20 weeks and above
2. All healthcare providers working in ANC units
3. All healthcare facilities of primary, secondary and tertiary level

Sample size estimation

Sample size estimation for pregnant women

The sample size of pregnant will be calculated by using formula of Cochran [21]. The proportion of pre-eclampsia of 9% [10], the margin of error of 5% and a Z-score value of 1.96 will be used. Therefore, the sample size will be 138 pregnant women.

Sample size estimation for the health care providers

The sample size of the health care providers will be calculated by using the formula Cochran [21]. The proportion of health care providers with knowledge on the management of pre-eclampsia of 11.8% [22], Z-score value of 1.96 and margin error of 5% will be used. Adding 10% of none response, the minimum sample size of 176 healthcare providers will be used.

Sample size estimation of healthcare facilities

The sample size will be calculated using a formula put forward by WHO which states that in assessing lower health facility levels, if there are 100 or fewer, you should study all of them. If there are more than 100 then take at least 30% [23]. In this regard, the total number of primary healthcare facilities in Zanzibar (PHCUs and PHCU+) is 154, therefore only 30% will be taken which is equal to 46 primary healthcare facilities. Moreover, the total number of PHCC is 4, district hospital is 2, the regional hospital is 1, and referral hospital is 1, therefore all of them will be included in the study. Therefore, the total healthcare facilities that will be included in this study will be 54.

Sampling procedure

Selection of pregnant wome[illegible]

Number of pregnant women per each selected healthcare facility level will be obtained using a proportionate sampling using a formula of Ni = (Ni/Nt)* n [24], whereby Ni = number of pregnant women needed in each facility, Ni = total number of pregnant women attending ANC in each selected facility per month, Nt = the total number of pregnant women attending ANC in all selected facilities per one month and n = the estimated sample size of pregnant women. Within healthcare facility, simple random sampling will be used to select study participants.

Selection of health care providers

Number of health care providers per each selected healthcare facility and from each professional cadre will be obtained using a proportionate sampling [24] whereby Ni = number of health care providers needed from each facility and from each cadre, Ni = total number of health care providers working in ANC in each cadre from each healthcare facility, Nt = total number of health care providers working in ANC in all selected facilities for each cadre, and n= the estimated sample size. Within the healthcare facility, all healthcare providers who met the inclusion criteria will be selected using simple random sampling.

Selection of healthcare facilities

All health centers (PHCCs), district hospitals, regional hospital and referral hospital will be included in this study. Moreover, systematic random sampling method will be used to select PHCUs whereby the sampling frame will be a total number of PHCUs and the kth interval will be calculated using the formula of $K^{th.}$ = N/n [25].

Measurement of Variables

Dependent Variables

Pre-eclampsia: This will be measured by the blood pressure reading of at least 140mmHg systolic and 90mmHg diastolic, measured on two occasions; 4 - 6 hours apart, accompanied by proteinuria of at least +1~~mg~~ on dipstick, and or severe headache or upper abdominal pain or blurred vision or light sensitivity. Nominal dichotomous scale will be used and the findings will be categorized into positive and negative for pre-eclampsia.

Management of pre-eclampsia: This refers to all treatment, measurements and tests done to a pregnant woman with signs of pre-eclampsia as per the MOHWZ guideline. This variable will be measured by 16 YES/NO items in ratio scale assessing whether management of pre-eclampsia was conducted as per the established guideline, which includes provision of

proper medications, monitoring of blood pressure and fetal heart rate, checking protein in urine and conducting liver and kidney function tests [4]. Five items will be for mild pre-eclampsia and eleven items for severe pre-eclampsia. One point will be awarded for the correct act carried out and 0 for the incorrect or missed acts. For mild pre-eclampsia a total score of 5 points will be regarded as proper management, and for severe pre-eclampsia a total score of 11 points will be regarded as proper management, less than that is improper management.

Status of meeting referral requirement: In this study, it is when the healthcare facility meets all required referral standards to refer a woman with signs of pre-eclampsia/eclampsia based on the recommended guidelines. This will be measured by 8 YES/NO items in nominal scale assessing whether the referrals requirements are met as per the established guideline. The tool will be adopted from WHO, (2013), and it includes assessing if the facility has an established clear referral pathway, provide pre-referral treatment to the patient, refer the patient accompanied by health care provider, has referral guideline and has ambulance.

Independent Variables

Knowledge of pre-eclampsia: This will be measured by 10 multiple choice questions in ratio scale. The standardized structured questionnaire will be adopted from Olaoye et al. [26] which has a Cronbach alpha was 0.72. One point will be awarded for the correct answer and zero point for the incorrect answer. Example of the question is 'What is the loading dose of magnesium sulfate?' The total score will be 10, and 75th percentile will be used to categories into those with adequate knowledge and inadequate knowledge. The same questionnaire will used to all categories of healthcare providers and the knowledge level will be presented for each category.

Skills for management of pre-eclampsia: This will be measured by 7 items in the form of task questions. The tool will be adopted from Jhpiego, 2017 [27]. Participants will be required to describe in details all the important steps in carrying out a certain procedure related to the management of pre-eclampsia. Example of the question is 'What are the steps for providing the loading dose of magnesium sulphate?' One point will be awarded for the complete mentioned steps and zero point for incomplete, incorrect or missed step. The total score will be 7 and 75th percentile will be used to categorize those with adequate skills against those with inadequate skills.

Availability of Equipment, tests and medication: These will be measured by 27 items on dichotomous nominal scale on the availability of well-functioning equipment necessary for the management of pre-eclampsia (14 items, e.g. working blood pressure machine, working fetoscope etc), the availability of essential medications (10 items, e.g. methyldopa and diazepam) and the availability of tests needed for the management of pre-eclampsia (3 items, eg. protein in urine test and kidney function test). The tool will be adopted from Maembe and Pembe, 2015 [28].

Background characteristics: The background characteristics of healthcare providers will be measured by variables from literature which will include years of working experience in ANC, working healthcare facility level, and ever attended training on the management of pre-eclampsia. The background characteristics of pregnant women will include number of ANC visits, having a history of pre-eclampsia, and having a history of using medication in the current pregnancy.

Data collection procedure:

Data will be collected within the selected healthcare facilities for the period of four weeks. The principal investigator and six research assistants who are registered nurses will collect the data. In this study, several methods of data collection methods will be used depending on the type of data needed to answer a particular study objective as follows:

Questionnaire: Self-administered questionnaire will be used to obtain data from healthcare providers for assessing their knowledge and skills on the management of pre-eclampsia while interviewer-administered questionnaire will be used to obtain data from pregnant women for assessing their background characteristics and signs and symptoms related to hypertensive disorders of pregnancy. Printed questionnaire for healthcare providers will be distributed in each healthcare facility and for pregnant women research assistant will be reading the questions and write down the response from participants.

Measurement method: This method will be used to get data on blood pressure readings of pregnant women to be used for making diagnosis of pre-eclampsia. In this method a research assistant will instruct a pregnant woman to relax at least ten minutes and sit comfortable in an up-right position with her back and arm supported and both legs flat on the floor. Moreover, a woman will be instructed to remove any tight clothes from around her arm. The appropriate

cuff size will be used where by cuff should encircle at least three-fourths of the circumference of arm in a 2 cm above elbow. The measurement will be taken by using digital blood pressure machine and will be taken twice at least four hours apart.

Chemical investigations method: Urinalysis with visual reagent strip, “dipstick” will be used to collect data on protein in urine. This data will be used together with other information to make diagnosis of pre-eclampsia. In this method a pregnant woman will be given a urine bottle and will be instructed on how to collect a clean-catch urine sample. The research assistant will dip the end of the dipstick into the urine sample and shake off any excess urine by tapping the dipstick on the side of the urine bottle. After thirty seconds a comparison of dipstick pad with the color chart on the dipstick container will be done to check for the presence of protein in urine.

Documentary review: This method will be used to obtain information on the management of pre-eclampsia whereby the principle investigator/research assistant will review the case files of patients diagnosed with pre-eclampsia, should it be mild or severe pre-eclampsia to look for management provided against the standard treatment guideline using pre-prepared checklist.

Observation method: This method will be used for assessing the availability of equipment, tests and medications related to the management of pre-eclampsia using a pre-prepared checklist. The same method will be used to assess whether the facility meets the referral standards. In this method, the principal investigator/research assistant will inspect the facility and record the available equipment, medication, tests, guidelines and referral practice records the pre-prepared checklists.

Data management plan

Data obtained from participants will be secured in safe computer with password that will be able to be accessed by the researcher’s team only and all safety data protocols of the University of Dodoma will be taking under consideration in order to secure participants information.

Data analysis plan

Data will be analyzed using SPSS software version 25. Data will be cleaned and checked for completeness before subjected to models of analysis. Descriptive statistics will be used to describe the proportion of pre-eclampsia, the frequency distribution of equipment, medication,

tests, referral standards, and knowledge and skills of health care providers on the management of pre-eclampsia. Chi-square test on the other hand will be used to show the association between pre-eclampsia and demographic characteristics of pregnant women. The same test will be used to show association between health care facility levels and management of pre-eclampsia, availability of equipment, test and medication, and availability of skilled and knowledgeable health care providers. In addition, chi square test will be used to show the association between knowledge and skills of health care providers on the management of pre-eclampsia and their background characteristics. In order to avoid hospital bias, the data collector will not be among nurses working in the selected facility, confidentiality from respondents will be assured and data will be analyzed by considering the level of healthcare facility, that is the findings for each healthcare facility level will be presented separately.

Ethical consideration and declaration

Ethical permission for this study was obtained from the University of Dodoma Research Ethics Committee (UDOM-REC) with reference number MA.84/261/02/218 and Zanzibar health research institute (ZAHRI) with a reference number ZAHREC/04/ST/JAN/2021/02. A written consent will be given to health care providers and pregnant women after explaining the purpose of the study and being told that their participation is voluntary and they can be able to withdrawal any time from the study.

Status and timeline of the study

The study is expected to be conducted for the period of four weeks, on the day of submission the status was in planning for data collection.

Discussion

Strength and limitation

The strength of this study is that it will involve all healthcare facilities in Zanzibar, form lower level to the highest level. This is important because the results that will be obtained will portray the true picture of the current status of pre-eclampsia management in healthcare facilities in Zanzibar. To the best of the authors knowledge, this will be the first study to be conducted in Zanzibar for auditing healthcare facilities in relation to pre-eclampsia management and referral. This information will help the Ministry of Health-Zanzibar to make proper decisions to strengthen healthcare services to pregnant women in terms of increasing the number of skilled health care providers, medication, equipment and tests. All these will contribute to minimize the delay of receiving appropriate management hence decreasing the burden of pre-

eclampsia/eclampsia complication. The limitation of this study design will be the difficulty to tally which patient who developed eclampsia was managed by which healthcare provider, therefore we will not be able to tell whether patient's complication was associated with poor service provided by unskilled healthcare provider or it is because of women failure to adhere to the management provided.

Dissemination plan

Finding obtained from this study will be disseminated to the University of Dodoma, to the Ministry of health Zanzibar and will be submitted for publishing in peer reviewed journal.

How amendments to the study, including termination, will be dealt with

If any amendments should be made to the study including termination of the study, this will be communicated to the journal auditoria and submit the changes or give reason for the termination.

Author's contributions

S.A.R designed the study and wrote the protocol for ethical approval. S.A.S reviewed and provide constructive inputs to improve this protocol for publication.

Acknowledgements

We acknowledge the support of Tasakhtaa Global Hospital-Zanzibar for the financial support to undertake master degree which lead to the production of this article.

Supporting information

Questionnaire for assessing knowledge and skills of healthcare providers

Questionnaire for assessing background characteristics of pregnant women

Checklist for assessing healthcare facilities for availability of medication, equipment and test

Checklist for assessing management of pre-eclampsia

Checklist for assessing the referral standard

References:

[1] ACOG Committee on Obstetric Practice, "Practice bulletin #33: diagnosis and management of preeclampsia and eclampsia," *Obstet. Gynecol.*, vol. 99, no. 1, pp. 159–167, 2002, doi: 10.1016/s0029-7844(01)01747-1.

[2] J. M. Roberts, "Contemporary Concepts of the Pathogenesis and Management of

Preeclampsia," no. June, pp. 3183–3186, 2014, doi: 10.1001/jama.287.24.3183.

[3] V. L. Bilano, E. Ota, T. Ganchimeg, R. Mori, and J. P. Souza, "Risk factors of pre-eclampsia/eclampsia and its adverse outcomes in low- and middle-income countries: A WHO secondary analysis," *PLoS One*, vol. 9, no. 3, pp. 1–9, 2014, doi: 10.1371/journal.pone.0091198.

[4] MHCDGEC, "Standard treatment guidelines & national essential medicines list, Tanzania mainland, 453. 2019, Retrieved from <https://apps.who.int/medicinedocs/documents/s23541en/s23541en.pdf> [5] L. Say et al., "Global causes of maternal death: A WHO systematic analysis," Lancet Glob. Heal., vol. 2, no. 6, pp. 323–333, 2014, doi: 10.1016/S2214-109X(14)70227-X.

[5] J. Wandabwa, P. Doyle, P. Kiondo, O. Campbell, N. Maconichie, and G. Welishe, "Risk factors for severe pre-eclampsia and eclampsia in Mulago Hospital, Kampala, Uganda.," *East Afr. Med. J.*, vol. 87, no. 10, pp. 415–424, 2010, doi: 10.4314/eamj.v87i10.

[6] K. R. Fingar, I. Mabry-Hernandez, Q. Ngo-Metzger, T. Wolff, C. A. Steiner, and A. Elixhauser, "Delivery Hospitalizations Involving Preeclampsia and Eclampsia, 2005–2014: Statistical Brief #222," *Healthc. Cost Util. Proj. Stat. Briefs*, pp. 1–26, 2006.

[7] A. N. Otieno, "Factors Contibuting To Adverse Outcomes Of Pre-Eclampsia Among Pregnant Women Attending Antenatal Clinics In Kibera Slums, Nairobi," 2012.

[8] A. S. Belay and T. Wudad, "Prevalence and associated factors of pre- eclampsia among pregnant women attending anti-natal care at Mettu Karl referal hospital , Ethiopia : cross-sectional study," pp. 1–8, 2019.

[9] I. O. Frank, J. M. Michael, R. Jenny, and O. Joseph, "Prevalence and risk factors for pre-eclampsia/eclampsia in Northern Tanzania," *J. Public Heal. Epidemiol.*, vol. 12, no. 2, pp. 78–85, 2020, doi: 10.5897/jphe2019.1143.

[10] N. Tufton and R. R. Patel, "Prevalence of hypertensive disorders in a prenatal clinic in Zanzibar," *Int. J. Gynecol. Obstet.*, vol. 112, no. 1, pp. 69–70, 2011, doi: 10.1016/j.ijgo.2010.09.005.

[11] L. Say *et al.*, "Global causes of maternal death: A WHO systematic analysis," *Lancet Glob. Heal.*, vol. 2, no. 6, pp. 323–333, 2014, doi: 10.1016/S2214-109X(14)70227-X.

[12] World Health Organization, "Trends in Maternal Mortality 2000 to 2017, pp. 16, 2019, [Online] Retrieved from <https://apps.who.int/iris/bitstream/handle/10665/327596/WHO-RHR-19.23-eng.pdf>

[13] MHCDGEC "Tanzania Demographic and Health Survey and Malaria Indicator Survey

2015-2016 - Final Report [FR321] | Enhanced Reader.” Retrieved from moz-extension://a6710f23-f161-476e-ae79-432076c7f649/enhanced-reader.html?openApp&pdf=https%3A%2F%2Fdhsprogram.com%2Fpubs%2Fpdf%2FFR321%2FFR321.pdf [14] D. A. M. Makuwani *et al.*, “Baseline Data on Trend of Maternal Mortality in Tanzania using Administrative Data and its Policy Implication. 2018 Report,” *Glob. J. Med. Res.*, vol. 20, no. 6, pp. 5–12, 2020, doi: 10.34257/gjmrkvol20is6pg5.

[15] MoHSW, "Zanzibar Health Management Information System Unit; Health Information Bulletin 2008", 2009, Retrieved from <https://mohz.go.tz/eng/health-bulletins/> [16] T. Herklots, L. Van Acht, T. Meguid, A. Franx, and B. Jacod, “Severe maternal morbidity in Zanzibar ’ s referral hospital : Measuring the impact of in- hospital care,” pp. 1–11, 2017.

[17] A. Barua, S. Mundle, H. Bracken, T. Easterling, and B. Winikoff, “Facility and personnel factors influencing magnesium sulfate use for eclampsia and pre-eclampsia in 3 Indian hospitals,” *Int. J. Gynecol. Obstet.*, vol. 115, no. 3, pp. 231–234, 2011, doi: 10.1016/j.ijgo.2011.07.016.

[18] O. Oguntunde *et al.*, “Factors influencing the use of magnesium sulphate in pre-eclampsia/eclampsia management in health facilities in Northern Nigeria: A mixed methods study,” *BMC Pregnancy Childbirth*, vol. 15, no. 1, pp. 1–8, 2015, doi: 10.1186/s12884-015-0554-8.

[19] Goverment of Zanzibar, “Management protocols for emergency obstetric and newborn care,” Zanzibar, 2019. Retrieved from <https://mohz.go.tz/wp-content/uploads/2021/01/Zanzibar-Reproductive-Maternal-Newborn.pdf> [20] NHSI (NHS Improvement) ACT Academy, “Quality, Service Improvement and Redesign Tools: A model for measuring quality care,” *Nhsi Act*, pp. 198–201, 2008.

[21] W. G. Cochran, “Double sampling,” *Cochran WG. Sampl. Tech. 3rd ed. New York John Wiley Sons, Inc*, pp. 327–358, 1977.

[22] S. Liljevik and E. S. V. B. Lohre, “Evaluation of knowledge and management practices of hypertension in pregnancy among health care workers in Moshi urban, Tanzania.,” pp. 1–41, 2012.

[23] WHO, "Monitoring Emergency Obstetric Care: a Handbook", 2009, Retrieved from http://whqlibdoc.who.int/publications/2009/9789241547734_eng.pdf

[24] R. Pandey and M. R. Verma, “Samples allocation in different strata for impact evaluation of developmental programme,” *Rev. Mat. Estat*, vol. 26, no. 4, pp. 103–112,

2008.
[25] R. Iachan, “Systematic Sampling: A Critical Review,” *Int. Stat. Rev. / Rev. Int. Stat.*, vol. 50, no. 3, p. 293, Dec. 1982, doi: 10.2307/1402499.
[26] T. Olaoye, O. O. Oyerinde, O. J. Elebuji, and O. Ologun, “Knowledge, Perception and Management of Pre-eclampsia among Health Care Providers in a Maternity Hospital,” *Int. J. MCH AIDS*, vol. 8, no. 2, pp. 80–88, 2019, doi: 10.21106/ijma.275.
[27] “Pre-eclampsia & Eclampsia (HMS PE&E) – Helping Mothers Survive.” https://hms.jhpiego.org/pre-eclampsia_eclampsia/ (accessed Dec. 29, 2021).
[28] L. E. Maembe and A. B. Pembe, “Managing pre-eclampsia and eclampsia in Dar es Salaam public health facilities: A focus on equipment, supplies, drugs and knowledge of healthcare workers,” *Tanzania Med. J.*, vol. 27, no. 1, pp. 43–54, 2015.

Figure Click here to access/download;Figure;figure 1.docx

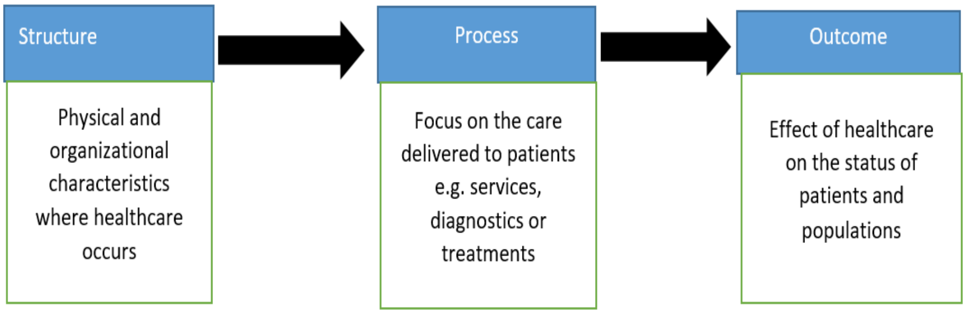

Figure 1: The Donabedian model for quality of care

Click here to access/download
Supporting Information - Compressed/ZIP File Archive
supporting information.zip

Auditing the Readiness of Healthcare Facilities in Antenatal Care Referral and Management of Pre-Eclampsia in Zanzibar- A Protocol for Cross Sectional Study

Salma A. Rashid[1¶*], Saada A. Seif[2¶]

[1] Department of Clinical Nursing, The University of Dodoma, Dodoma, Tanzania

[2]Department of Nursing Management and Education, The University of Dodoma, Dodoma, Tanzania

Funding: No fund received for this study

Competing interest: There is no competing interest to declare

Data availability: "Deidentified research data will be made publicly available when the study is completed and published"~~Data will be available upon request~~

[*]Corresponding author
Email: sallyrasheed.sr@gmail.com

[¶]These authors contributed equally to this work

Abstract

Hypertensive disorders of pregnancy reported as the second leading root of maternal morbidity and mortality in Zanzibar. Evidences show that majority of pregnant women in Zanzibar are referred late from antenatal clinics and most end up with eclampsia. The objective of this study is ~~therefore~~ to determine the prevalence of pre-eclampsia, and to assess the readiness of health care facilities in relation to pre-eclampsia referral and management in Zanzibar.

This is a descriptive cross-sectional study that will involve a total of 54 healthcare facilities, 176 health care providers and 138 pregnant women with gestation age of 20 weeks and above. Health care facilities will be selected by systematic random sampling, and simple random sampling will be used to select health care providers and pregnant women. Protein in urine test and measurement of blood pressure will be conducted ~~done~~ for the diagnosis of pre-eclampsia. Healthcare facilities will be assessed if they meet the requirement for referral ~~standards~~ and for the availability of equipment and supplies, medications and lab-tests related to the management of pre-eclampsia using a pre-prepared checklist. Questionnaire will be used to determine the proportion of healthcare providers with knowledge and skill on the management of pre-eclampsia within each facility, and to obtain background characteristics information from pregnant women, while patients case files in each facility will be reviewed to assess the management of pre-eclampsia using a pre-prepared checklist. Data will be analysis using SPSS software version 25. Descriptive statistics will be used to describe the frequency distribution of the study variables and results will be presented in frequency and percentage. Chi square test will be used to describe the relationship between variables and the p-value of < 0.05 will be regarded as statistically significant difference.

~~Ethical consideration~~

~~This protocol was approved by University of Dodoma Research Ethics Committee (UDOM-REC) with reference number MA.84/261/01/92 and Zanzibar health research institute (ZAHRI) with reference number ZAHREC/04/ST/JAN/2021/02. Written informed consent will be obtained from healthcare providers and pregnant women.~~

Keywords:

Pre-eclampsia, eclampsia, healthcare facilities readiness, antenatal care referral, management of pre-eclampsia, Zanzibar.

Introduction

Pre-eclampsia is can be defined as a pregnancy complication characterized by high blood pressure Blood Pressure (BP) of at least 140mmHg systolic pressure and 90mmHg diastolic pressure measured on two occasions, 4 - 6 hours apart, accompanied by proteinuria of at least 300 mg per 24 hours, or at least +1mg on dipstick testeding after 20 weeks of gestation age and or with swelling of lower limbs, severe headache and or upper abdominal pain and or blurred vision or light sensitivity. While eclampsia is the existence of a new-onset of grand mal-seizure in women with pre-eclampsia [1]. The pathogenesis of pre-eclampsia is not known though it is associated with abnormal placentation at the beginning of pregnancy, while hypertension and proteinuria occurred in the second half of pregnancy [2]. The risk factors like obesity, chronic hypertension, diabetics, nulliparous, and twinning pregnancy have been shown to increase the likelihood of developing pre-eclampsia to pregnant women [3].

Pre-eclampsia is categorized as mild and severe, the mild one is diagnosed when BPblood pressure is greater than 140 systolic or 90 diastolic and 0.3mg of protein is collected in a 24-hour urine sample, or persistent of +1mg protein measurement in urine dipstick. While severe one is diagnosed when the blood pressure BP is greater than 160/110 mmHg (especially diastolic ≥110 mmHg), proteinuria of 3+mg or greater or 1mg or greater in 24hours, with severe headache, epigastric pain, blurring of vision and vomiting[4]. The prognosis from mild to severe can be fast and unpredictable if left untreated.

Researches show that Pre-Eclampsia/Eclampsia (PE/E) is among the primary cause of maternal and neonatal morbidity and mortality. It is reported to affect 2- 8% of pregnancies globally, and it is associated with 10- 15% of direct maternal deaths and up to 25% of stillbirths and newborn deaths in developing countries [5], [6]. Tanzania is reported to have 556 maternal death per 100,000 live birth [7], and eclampsia related complications accounts for 18.9% to maternal death [8]. In Zanzibar, the reported maternal mortality is 155 death per 100,000 live birth [9], and severe pre-eclampsia was found to contribute to 25.8% of maternal morbidity and 21.8%of maternal mortality [10].

The prevalence of pre-eclampsia/eclampsia ~~PE/E~~ is reported to be much higher in developing countries which ranges from 1.8 to 16.7% [5], while in developed countries like United States of America, the pre-eclampsia prevalence ranges from 3-6% [6]. In East African countries, the prevalence of p~~P~~re-eclampsia ranges from 6.1% in Kenya [7] to 12.2% in Ethiopia [8]. The recent study done in Tanzania showed the prevalence of severe pre-eclampsia is 4.2% [9] while in Zanzibar the prevalence of pre-eclampsia reported in the year 2011 was 9% [10]. Therefore, there is still need to determine the current prevalence of pre-eclampsia in the country to understand its trend.

Researches show that pre-eclampsia/eclampsia is among the primary cause of maternal and neonatal morbidity and mortality. It is reported to affect 2–8% of pregnancies globally, and it is associated with 10–15% of direct maternal deaths and up to 25% of stillbirths and newborn deaths in developing countries [11], [12]. Tanzania is reported to have 556 maternal deaths per 100,000 live birth [13], and eclampsia related complications accounts for 18.9% to maternal deaths [14]. In Zanzibar, the report of 2008 showed that maternal mortality was 155 deaths per 100,000 live birth [15], while the report of 2017 showed that severe pre-eclampsia was found to contribute to 25.8% of maternal morbidity and 21.8% of maternal mortality [16].

Effective management of pre-eclampsia/eclampsia can be possible by following the WHO guideline ~~The World Health Organization (WHO) developed a guideline~~ of Antenatal Care (ANC) screening for early detection of signs of pre-eclampsia as well as the following the guideline for pharmacological management ~~to prevent PE/E. The guideline also includes the proper management of pre-eclampsia~~ and meet the specified requirement for referral. ~~standards.~~ Poor adherence to the~~i~~se guidelines can result in poor management of pregnant women with signs of pre-eclampsia and thus may contributes to increasing of maternal morbidity and mortality related to pre-eclampsia/eclampsia. ~~Therefor it is very important to assess how much does this guideline is followed in different healthcare facility levels.~~

~~According to the WHO guideline, screening of PE/E requires simple tools like sphygmomanometer and stethoscope for monitoring blood pressure, and albustix for checking protein in the urine. All these must be available in healthcare facilities of all levels. In addition, tests like renal function test, liver function test, and tests to evaluate platelets counts which are~~

mostly needed in the severe form of PE/E must to be available in the higher healthcare facility levels [1]. The definitive treatment of preeclampsia/eclampsia is delivery of the baby, but the decision is based on the severity of pre-eclampsia, gestational age, maternal and fetal condition [17], while the conservative treatment of pre-eclampsia involves control of BP and prevention of seizure. All treatment aiming at minimizing pregnancy related complications, minimizing prematurity and promote maternal and infant survival [17]. Therefore, understanding the availability of screening tools and how pregnant women are managed at different healthcare facility levels is very crucial in developing intervention aiming at reducing maternal and neonatal morbidity and mortality.

The pharmacological agents used for controlling high BP or a diastolic blood pressure of less than 90 mmHg and systolic blood pressure of less than 140mmHg are methyldopa, nifedipine, hydralazine, and labetalol. When the blood pressure reaches 160mmHg systolic or 110mmHg diastolic, the woman becomes at high risk of developing complications, and thus a rapid control of blood pressure is needed. Therefore intravenous labetalol and hydralazine are highly recommended [4]. For prevention of pre-eclampsia, the WHO recommends the use of calcium supplement at doses of 1.5–2.0mg elemental calcium/day in areas where dietary calcium intake is low to all women especially those at high risk of developing pre-eclampsia. Moreover, a low-dose acetylsalicylic acid (aspirin, 75 mg/day) is recommended to women at high risk of developing pre-eclampsia [18].

Various factors that affect the management of pre-eclampsia/eclampsia have been identified, and these include unavailability of medication and supply and lack of healthcare providers with adequate knowledge and skills for the management of pre-eclampsia. All these may result in failure to early detect, diagnose and providing proper treatment of pre-eclampsia [17], [18]. Therefore, assessing these factors in each healthcare facility levels is very important step for enhancing good decision making for the distribution of medical supplies related to the management of pre-eclampsia/eclampsia PE/E at different healthcare facility levels.

Several measures have been taken by the Tanzanian government to reduce the rate of PE/E which include sensitizing the community on effective utilization of ANC services, implementation of Focused Antenatal Care (FANC), facilitating early detection of danger signs, screening of blood

~~pressure and protein in urine testing. From these efforts, data show that 98% of pregnant women received ANC, 87% of pregnant women have their blood sample taken, 71% have their blood pressure checked and 60% have their urine sample collected [21].~~

~~Specifically, in Zanzibar, t~~The Ministry of Health Zanzibar adopted the WHO guideline and developed the ‘Management Protocols of Obstetric and Newborn Care’ which is to be uniformly followed at all healthcare facility levels. In addition, health care providers are provided with training on focused antenatal care which include the use~~s~~ of these~~is~~ guidelines. According to this protocol all pregnant women attending ANC must be screened for pre-eclampsia/eclampsia~~PE/E~~, and those showing signs of pre-eclampsia/eclampsia~~PE/E~~, must be appropriately managed, and those presented with severe pre-eclampsia at lower healthcare facility levels, must be referred to the next level immediately [19]. However, evidence showed that majority of pregnant women in Zanzibar are referred late and most ends up with eclampsia (25.8%)[16].

Hence the questions come of whether all levels of healthcare facilities in Zanzibar are prepared enough in terms of availability of the required equipment, medications, adherence to management and referral guidelines as well as the availability of healthcare providers with knowledge and skills for the management of pre-eclampsia/eclampsia~~PE/E~~. On this ground, this research therefore intends to assess the prevalence of pre-eclampsia and the healthcare facilities readiness in relation to ANC referral and management of pre-eclampsia in Zanzibar.

Study model: Donabedian model for measuring quality of care

This study will be guided by model for measuring quality care by Donabedian (Fig. 1) [20]. This model has three approaches to evaluation of the quality of care, which are structure, process, and outcome. The structure which is also known as input measure entails the characteristics of the healthcare facility ~~provider~~ such as staff to patient ratio and operating times and hours of the services. Process measures reflect the way systems work to deliver the desired outcome, and the outcome measure reflects the impact on the patient and demonstrates the result of improvement and whether it has ultimately achieved the aim(s) set [20].

The current study will adopt all approaches as specified by the model as follows: The structure measures will be the availability of resources in the health~~-~~care facilities like skilled and knowledgeable health~~-~~care providers, availability of guidelines, availability of required tests,

resources and equipment, on job training, availability of ambulance, and availability of medication. The process measure will be the adherence to the guideline on the treatment of pre-eclampsia/eclampsia and adherence of guidelines in making the referral to women with signs of severe pre-eclampsia. The outcome measure will be the number of emergency admissions due to severe pre-eclampsia and the number of patients with eclampsia.

Methods

Study objectives

The general objective of this study is to determine the proportion of pre-eclampsia and to assess the readiness of healthcare facilities in relation to pre-eclampsia antenatal care referral and its management in Zanzibar.

The Specific objectives are:

1. To determine the prevalence of pre-eclampsia among pregnant women attending ANC.
2. To assess the availability of equipment, medication and tests related to the management of pre-eclampsia among all healthcare facility levels.
3. To determine proportion of health care providers working in ANC with adequate knowledge on the management of pre-eclampsia
4. To determine proportion of health care providers working in ANC with adequate skills on the management of pre-eclampsia
5. To assess the management of pre-eclampsia in all healthcare facility levels
6. To assess the status of meeting the referral requirement ~~standards~~ among healthcare facilities of lower levels

Study design

A descriptive cross-sectional design using a quantitative approach will be used.

Study area

This study will be conducted in Zanzibar which is united with and a part of Tanzania. The study setting will be healthcare facilities of all levels in Zanzibar. The healthcare delivery system in Zanzibar is organized into three levels which are the primary level, secondary and tertiary level. The primary level is the lowest level which includes Primary Health Care Unit (PHCU), Primary Health Care Unit Plus (PHCU +) and Primary Health Care Center or Cottage (PHCC). In total, there are 122 PHCU, 34 PHCU+, and 4 PHCC. Primary health care units provide primary health care services and all ANC services including screening of pre-eclampsia, while the PHCU+

provides additional services such as delivery, dental, laboratory, and pharmaceutical services while the PHCCs provide the same services as PHCU+ with the addition of inpatient and X-ray services.

The secondary level which is the middle level of health care delivery includes district hospitals and the regional hospitals. There are two district hospitals and one regional hospital. District hospitals provide second-line referral services including basic surgery, and regional hospitals provide referral points for all district hospitals. The highest level of health care delivery is the tertiary level or called the referral hospital. There is only one referral hospital in Zanzibar which is Mnazi Mmoja National Hospital which provides referral services from the district and regional hospitals.

~~According to the guideline provided by the Ministry of Health Zanzibar (Management Protocols of Obstetric and Newborn Care), all pregnant women attending ANC at any healthcare facility level have to be screened for pre-eclampsia, and those with signs of pre-eclampsia should managed according the guideline. Moreover, all women with signs of severe pre-eclampsia seen at the PHCU and PHCU+ have to be treated with pre-referral treatment and referred immediately as per the guideline procedure to the nearest PHCC or district hospital depending on the accessibility of the health facility, while most complicated cases have to be referred to tertiary hospital for further management.~~

Study Population

This study will involve three types of study populations**.**

1. All pregnant women attending ANC with gestation age of 20 weeks and above
2. All healthcare providers working in ANC units
3. All healthcare facilities of primary, secondary and tertiary level

Sample size estimation

Sample size estimation for pregnant women

The sample size of pregnant will be calculated by using formula of Cochran [21]. The proportion of pre-eclampsia of 9% [10], the margin of error of 5% and a Z-score value of 1.96 will be used. Therefore, the sample size will be 138 pregnant women.

Sample size estimation for the health care providers

The sample size of the health care providers will be calculated by using the formula Cochran [21]. The proportion of health care providers with knowledge on the management of pre-eclampsia of 11.8% [22], Z-score value of 1.96 and margin error of 5% will be used. Adding 10% of none response, the minimum sample size of 176 healthcare providers will be used.

Sample size estimation of healthcare facilities

The sample size will be calculated using a formula put forward by WHO which states that in assessing lower health facility levels, if there are 100 or fewer, you should study all of them. If there are more than 100 then take at least 30% [23]. In this regard, the total number of primary healthcare facilities in Zanzibar (PHCUs and PHCU+) is 154, therefore only 30% will be taken which is equal to 46 primary healthcare facilities. Moreover, the total number of PHCC is 4, district hospital is 2, the regional hospital is 1, and referral hospital is 1, therefore all of them will be included in the study. Therefore, the total healthcare facilities that will be included in this study will be 54.

Sampling procedure

Selection of pregnant women

Number of pregnant women per each selected healthcare facility level will be obtained using a proportionate sampling using a formula of Ni = (Ni/Nt)* n [24], whereby Ni = number of pregnant women needed in each facility, Ni = total number of pregnant women attending ANC in each selected facility per month, Nt = the total number of pregnant women attending ANC in all selected facilities per one month and n = the estimated sample size of pregnant women. Within healthcare facility, simple random sampling will be used to select study participants.

Selection of health care providers

Number of health care providers per each selected healthcare facility and from each professional cadre will be obtained using a proportionate sampling [24] whereby Ni = number of health care providers needed from each facility and from each cadre, Ni = total number of health care providers working in ANC in each cadre from each healthcare facility, Nt = total number of health care providers working in ANC in all selected facilities for each cadre, and n= the estimated sample size. Within the healthcare facility, all healthcare providers who met the inclusion criteria will be selected using simple random sampling.

Selection of healthcare facilities

All health centers (PHCCs), district hospitals, regional hospital and referral hospital will be included in this study. Moreover, systematic random sampling method will be used to select PHCUs whereby the sampling frame will be a total number of PHCUs and the kth interval will be calculated using the formula of $K^{th.} = N/n$ [25].

~~Variables definition~~

~~**Pre-eclampsia:** In this study refers to pregnancy complication characterized by high blood pressure after 20 weeks of pregnancy plus excess protein in urine and or severe headache and or upper abdominal pain and or blurred vision or light sensitivity.~~

~~**Management of pre-eclampsia:** In this study refers to all treatment provided, measurements and tests done to a pregnant woman with signs of pre-eclampsia as per the MOHWZ guideline.~~

~~**Meeting referral standards**: in this study is when the healthcare facility meets all required referral standards to refer a woman with signs of pre-eclampsia/eclampsia based on the recommended guidelines.~~

Measurement of Variables

Dependent Variables

Pre-eclampsia: This will be measured by the blood pressure reading of at least 140mmHg systolic and 90mmHg diastolic, measured on two occasions; 4 - 6 hours apart, accompanied by proteinuria of at least +1mg on dipstick, and or severe headache or upper abdominal pain or blurred vision or light sensitivity. Nominal dichotomous scale will be used and the findings will be categorized into positive and negative for pre-eclampsia.

Management of pre-eclampsia: This refers to all treatment, measurements and tests done to a pregnant woman with signs of pre-eclampsia as per the MOHWZ guideline. This variable will be measured by 16 YES/NO items in ratio scale assessing whether management of pre-eclampsia was conducted as per the established guideline, which includes provision of proper medications, monitoring of blood pressure~~BP~~ and fetal heart rate, checking protein in urine and conducting liver and kidney function tests~~. The tool will be developed from the guideline~~ [4]. Five items will be for

mild pre-eclampsia and eleven items for severe pre-eclampsia. One point will be awarded for the correct act carried out and 0 for the incorrect or missed acts. For mild pre-eclampsia a total score of 5 points will be regarded as proper management, and for severe pre-eclampsia a total score of 11 points will be regarded as proper management, less than that is improper management.

Status of meeting referral requirement~~standards~~: In this study , it is when the healthcare facility meets all required referral standards to refer a woman with signs of pre-eclampsia/eclampsia based on the recommended guidelines. This will be measured by 8 YES/NO items in nominal scale assessing whether the referrals requirements are met as per the established guideline. The tool will be adopted from WHO, (2013), and it includes assessing if the facility has an established clear referral pathway, provide pre-referral treatment to the patient, refer the patient accompanied by health care provider, has referral guideline and has ambulance.

Formatted: Font: Font color: Black, Kern at 12 pt

Independent Variables

Knowledge of pre-eclampsia: This will be measured by 10 multiple choice questions in ratio scale. The standardized structured questionnaire will be adopted from Olaoye et al. [26] which has a Cronbach alpha was 0.72. One point will be awarded for the correct answer and zero point for the incorrect answer. Example of the question is 'What is the loading dose of magnesium sulfate?' The total score will be 10, and 75~~50~~th percentile will be used to categories into those with adequate knowledge and inadequate knowledge. The same questionnaire will used to all categories of healthcare providers and the knowledge level will be presented for each category.

Skills for management of pre-eclampsia: This will be measured by 7 items in the form of task questions. The tool will be adopted from Jhpiego, 2017 [27]. ~~and will be little modified to suit the current study. A p~~Participants will be required to describe in details ~~mention~~ all the important steps in carrying out a certain procedure related ~~in relation~~ to the management of pre-eclampsia. Example of the question is 'What are the steps for providing the loading dose of magnesium sulphate?' One point will be awarded for the complete mentioned steps and zero point for incomplete, incorrect or missed step. The total score will be 7 and ~~50~~75th percentile will be used to categorize those with adequate skills against those with inadequate skills.

Availability of Equipment, tests and medication: These will be measured by 27 items on dichotomous nominal scale on the availability of well-functioning equipment necessary for the management of pre-eclampsia (14 items, e.g. working blood pressure~~BP~~ machine, working fetoscope etc), the availability of essential medications (10 items, e.g. methyldopa and diazepam) and the availability of tests needed for the management of pre-eclampsia (3 items, eg. ~~p~~Protein in urine test and kidney function test). The tool will be adopted from Maembe and Pembe, 2015 [28].

Background characteristics: The background characteristics of healthcare providers will be measured by variables from literature which will include years of working experience in ANC, working healthcare facility level, and ever attended training on the management of pre-eclampsia. The background characteristics of pregnant women will include number of ANC visits, ~~the~~ having a history of pre-eclampsia, and having a history of using medication in the current pregnancy.

Data collection procedure:

Data will be collected within the selected healthcare facilities for the period of four weeks. The principal investigator and six research assistants who are registered nurses will collect the data. In this study, several methods of data collection methods will be used depending on the type of data needed to answer a particular study objective as follows:

~~Interviewer-administered q~~Questionnaire: Self-administered questionnaire ~~This method~~ will be used to obtain data from healthcare providers for assessing their knowledge and skills on the management of pre-eclampsia while interviewer-administered questionnaire~~. The same method~~ will be used to obtain data from pregnant women for assessing their background characteristics and signs and symptoms related to hypertensive disorders of pregnancy~~.~~ Printed questionnaire for healthcare providers will be distributed in each healthcare facility and for pregnant women research assistant will be reading the questions and write down the response from participants.

Measurement method: This method will be used to get data on blood pressure readings of pregnant women to be used for making diagnosis of pre-eclampsia. In this method a research assistant will instruct a pregnant woman to relax at least ten minutes and sit comfortable in an up-

right position with her back and arm supported and both legs flat on the floor. Moreover, a woman will be instructed to remove any tight clothes from around her arm. The appropriate cuff size will be used where by cuff should encircle at least three-fourths of the circumference of arm in a 2 cm above elbow. The measurement will be taken by using digital blood pressure machine and will be taken twice at least four hours apart.

Chemical investigations method: Urinalysis with visual reagent strip, “dipstick” will be used to collect data on protein in urine. This data will be used together with other information to make diagnosis of pre-eclampsia. In this method a pregnant woman will be given a urine bottle and will be instructed on how to collect a clean-catch urine sample. The research assistant will dip the end of the dipstick into the urine sample and shake off any excess urine by tapping the dipstick on the side of the urine bottle. After thirty seconds a comparison of dipstick pad with the color chart on the dipstick container will be done to check for the presence of protein in urine.

Documentary review: This method will be used to obtain information on the management of pre-eclampsia whereby the principle investigator/research assistant will review the case files of patients diagnosed with pre-eclampsia, should it be mild or severe pre-eclampsia to look for management provided against the standard treatment guideline using pre-prepared checklist.

Observation method: This method will be used for assessing the availability of equipment, tests and medications related to the management of pre-eclampsia using a pre-prepared checklist. The same method will be used to assess whether the facility meets the referral standards. In this method, the principal investigator/research assistant will inspect the facility and record the available equipment, medication, tests, guidelines and referral practice records the pre-prepared checklists.

Data management plan

Data obtained from participants will be secured in safe computer with password that will be able to be accessed by the researcher’s team only and all safety data protocols of the University of Dodoma will be taking under consideration in order to secure participants information.

Data analysis plan

Data will be analyzed using SPSS software version 25. Data will be cleaned and checked for completeness before subjected to models of analysis. Descriptive statistics will be used to describe the proportion of pre-eclampsia, the frequency distribution of equipment, medication, tests, referral standards, and knowledge and skills of health care providers on the management of pre-eclampsia. Chi-square test on the other hand will be used to show the association between pre-eclampsia and demographic characteristics of pregnant women. The same test will be used to show association between health care facility levels and management of pre-eclampsia, availability of equipment, test and medication, and availability of skilled and knowledgeable health care providers. In addition, chi square test will be used to show the association between knowledge and skills of health care providers on the management of pre-eclampsia and their background characteristics. In order to avoid hospital bias, the data collector will not be among nurses working in the selected facility, confidentiality from respondents will be assured and data will be analyzed by considering the level of healthcare facility, . Tthat is the findings for each healthcare facility level will be presented analyzed separately.

Ethical consideration and declaration

Ethical permission for this study was obtained from the University of Dodoma Research Ethics Committee (UDOM-REC) with reference number MA.84/261/02/218 and Zanzibar health research institute (ZAHRI) with a reference number ZAHREC/04/ST/JAN/2021/02. A written consent will be given to health care providers and pregnant women after explaining the purpose of the study and being told that their participation is voluntary and they can be able to withdrawal any time from the study. Authors declare that this is an original work and have no interest to declare.

Status and timeline of the study

The study is expected to be conducted for the period of four weeks, on the day of submission the status was in planning for data collection.

Discussion

Strength and limitation

The strength of this study is that it will involve all healthcare facilities in Zanzibar, form lower level to the highest level. This is important because the results that will be obtained will portray the true picture of the current status of pre-eclampsia management in healthcare facilities in Zanzibar. To the best of the authors~~our~~ knowledge, this will be the first study to be conducted in Zanzibar for auditing healthcare facilities in relation to pre-eclampsia management and referral. This information will help the Ministry of Health-Zanzibar to make proper decisions to strengthen healthcare services to pregnant women in terms of increasing the number of skilled health care providers, medication, equipment and tests. All these will contribute to minimize the delay of receiving appropriate management hence decreasing the burden of pre-eclampsia/eclampsia complication. The limitation of this study design will be the difficulty to tally which patient who developed eclampsia was managed by which healthcare provider, therefore we will not be able to tell whether patient's complication was associated with poor service provided by unskilled healthcare provider or it is because of women failure to adhere to the management provided.

Dissemination plan

Finding obtained from this study will be disseminated to the University of Dodoma, to the Ministry of health Zanzibar and will be submitted for publishing in peer reviewed journal.

How amendments to the study, including termination, will be dealt with

If any amendments should be made to the study including termination of the study, this will be communicated to the journal auditoria and submit the changes or give reason for the termination.

Author's contributions

S.A.R designed the study and wrote the protocol for ethical approval. S.A.S reviewed and provide constructive inputs to improve this protocol for publication.

Acknowledgements

We acknowledge the support of Tasakhtaa Global Hospital-Zanzibar for the financial support to undertake master degree which lead to the production of this article.

Supporting information

Questionnaire for assessing knowledge and skills of healthcare providers

Questionnaire for assessing background characteristics of pregnant women

Checklist for assessing healthcare facilities for availability of medication, equipment and test

Checklist for assessing management of pre-eclampsia

Checklist for assessing the referral standard

References:

[1] ACOG Committee on Obstetric Practice, “Practice bulletin #33: diagnosis and management of preeclampsia and eclampsia,” *Obstet. Gynecol.*, vol. 99, no. 1, pp. 159–167, 2002, doi: 10.1016/s0029-7844(01)01747-1.

[2] J. M. Roberts, “Contemporary Concepts of the Pathogenesis and Management of Preeclampsia,” no. June, pp. 3183–3186, 2014, doi: 10.1001/jama.287.24.3183.

[3] V. L. Bilano, E. Ota, T. Ganchimeg, R. Mori, and J. P. Souza, “Risk factors of pre-eclampsia/eclampsia and its adverse outcomes in low- and middle-income countries: A WHO secondary analysis,” *PLoS One*, vol. 9, no. 3, pp. 1–9, 2014, doi: 10.1371/journal.pone.0091198.

[4] MHCDGEC, "Standard treatment guidelines & national essential medicines list, Tanzania mainland, 453. 2019, Retrieved from <https://apps.who.int/medicinedocs/documents/s23541en/s23541en.pdf> [5] L. Say et al., “Global causes of maternal death: A WHO systematic analysis,” Lancet Glob. Heal., vol. 2, no. 6, pp. 323–333, 2014, doi: 10.1016/S2214-109X(14)70227-X. ~~N. STG, “STANDARD TREATMENT GUIDELINES & NATIONAL ESSENTIAL MEDICINES LIST TANZANIA MAINLAND,” TANZANIA, 2017.~~

[5] J. Wandabwa, P. Doyle, P. Kiondo, O. Campbell, N. Maconichie, and G. Welishe, “Risk factors for severe pre-eclampsia and eclampsia in Mulago Hospital, Kampala, Uganda.,” *East Afr. Med. J.*, vol. 87, no. 10, pp. 415–424, 2010, doi: 10.4314/eamj.v87i10.

[6] K. R. Fingar, I. Mabry-Hernandez, Q. Ngo-Metzger, T. Wolff, C. A. Steiner, and A. Elixhauser, “Delivery Hospitalizations Involving Preeclampsia and Eclampsia, 2005–2014: Statistical Brief #222,” *Healthc. Cost Util. Proj. Stat. Briefs*, pp. 1–26, 2006.

[7] A. N. Otieno, “Factors Contibuting To Adverse Outcomes Of Pre-Eclampsia Among Pregnant Women Attending Antenatal Clinics In Kibera Slums, Nairobi,” 2012.

[8] A. S. Belay and T. Wudad, “Prevalence and associated factors of pre- eclampsia among pregnant women attending anti-natal care at Mettu Karl referal hospital , Ethiopia : cross-sectional study,” pp. 1–8, 2019.

[9] I. O. Frank, J. M. Michael, R. Jenny, and O. Joseph, “Prevalence and risk factors for pre-eclampsia/eclampsia in Northern Tanzania,” *J. Public Heal. Epidemiol.*, vol. 12, no. 2, pp. 78–85, 2020, doi: 10.5897/jphe2019.1143.

[10] N. Tufton and R. R. Patel, “Prevalence of hypertensive disorders in a prenatal clinic in Zanzibar,” *Int. J. Gynecol. Obstet.*, vol. 112, no. 1, pp. 69–70, 2011, doi: 10.1016/j.ijgo.2010.09.005.

[11] L. Say *et al.*, “Global causes of maternal death: A WHO systematic analysis,” *Lancet Glob. Heal.*, vol. 2, no. 6, pp. 323–333, 2014, doi: 10.1016/S2214-109X(14)70227-X.

[12] World Health Organization, "Trends in Maternal Mortality 2000 to 2017, pp. 16, 2019, [Online] Retrieved from <https://apps.who.int/iris/bitstream/handle/10665/327596/WHO-RHR-19.23-eng.pdf> ~~M. WHO, Mortality, *2000 to 2017*. 2017.~~

[13] MHCDGEC “Tanzania Demographic and Health Survey and Malaria Indicator Survey 2015-2016 - Final Report [FR321] | Enhanced Reader.” Retrieved from moz-extension://a6710f23-f161-476e-ae79-432076c7f649/enhanced-reader.html?openApp&pdf=https%3A%2F%2Fdhsprogram.com%2Fpubs%2Fpdf%2FFR321%2FFR321.pdf ~~“Tanzania Demographic and Health Survey and Malaria Indicator Survey 2015-2016 - Final Report [FR321] | Enhanced Reader.” .~~

[14] D. A. M. Makuwani *et al.*, “Baseline Data on Trend of Maternal Mortality in Tanzania using Administrative Data and its Policy Implication. 2018 Report,” *Glob. J. Med. Res.*, vol. 20, no. 6, pp. 5–12, 2020, doi: 10.34257/gjmrkvol20is6pg5.

[15] MoHSW, "Zanzibar Health Management Information System Unit; Health Information Bulletin 2008", 2009, Retrieved from <https://mohz.go.tz/eng/health-bulletins/> ~~MoHASWZ, “Ministry of Health & Social Welfare , Zanzibar Health Management Information System Unit Health Information Bulletin 2008,” 2018.~~

[16] T. Herklots, L. Van Acht, T. Meguid, A. Franx, and B. Jacod, “Severe maternal morbidity in Zanzibar ’ s referral hospital : Measuring the impact of in- hospital care,” pp. 1–11,

2017.

[17] A. Barua, S. Mundle, H. Bracken, T. Easterling, and B. Winikoff, "Facility and personnel factors influencing magnesium sulfate use for eclampsia and pre-eclampsia in 3 Indian hospitals," *Int. J. Gynecol. Obstet.*, vol. 115, no. 3, pp. 231–234, 2011, doi: 10.1016/j.ijgo.2011.07.016.

[18] O. Oguntunde *et al.*, "Factors influencing the use of magnesium sulphate in pre-eclampsia/eclampsia management in health facilities in Northern Nigeria: A mixed methods study," *BMC Pregnancy Childbirth*, vol. 15, no. 1, pp. 1–8, 2015, doi: 10.1186/s12884-015-0554-8.

[19] Goverment of Zanzibar, "Management protocols for emergency obstetric and newborn care," Zanzibar, 2019. Retrieved from <https://mohz.go.tz/wp-content/uploads/2021/01/Zanzibar-Reproductive-Maternal-Newborn.pdf> ~~goverment of Zanzibar, "management protocols for emergency obstetric and newborn care," Zanzibar, 2019.~~

[20] NHSI (NHS Improvement) ACT Academy, "Quality, Service Improvement and Redesign Tools: A model for measuring quality care," *Nhsi Act*, pp. 198–201, 2008.

[21] W. G. Cochran, "Double sampling," *Cochran WG. Sampl. Tech. 3rd ed. New York John Wiley Sons, Inc*, pp. 327–358, 1977.

[22] S. Liljevik and E. S. V. B. Lohre, "Evaluation of knowledge and management practices of hypertension in pregnancy among health care workers in Moshi urban, Tanzania.," pp. 1–41, 2012.

[23] WHO, "Monitoring Emergency Obstetric Care: a Handbook", 2009, Retrieved from http://whqlibdoc.who.int/publications/2009/9789241547734_eng.pdf~~WHO, "Monitoring Emergency Obsetric a Handbook," 2009.~~

[24] R. Pandey and M. R. Verma, "Samples allocation in different strata for impact evaluation of developmental programme," *Rev. Mat. Estat*, vol. 26, no. 4, pp. 103–112, 2008.

[25] R. Iachan, "Systematic Sampling: A Critical Review," *Int. Stat. Rev. / Rev. Int. Stat.*, vol. 50, no. 3, p. 293, Dec. 1982, doi: 10.2307/1402499.

[26] T. Olaoye, O. O. Oyerinde, O. J. Elebuji, and O. Ologun, "Knowledge, Perception and Management of Pre-eclampsia among Health Care Providers in a Maternity Hospital," *Int. J. MCH AIDS*, vol. 8, no. 2, pp. 80–88, 2019, doi: 10.21106/ijma.275.

[27] "Pre-eclampsia & Eclampsia (HMS PE&E) – Helping Mothers Survive." https://hms.jhpiego.org/pre-eclampsia_eclampsia/ (accessed Dec. 29, 2021).
[28] L. E. Maembe and A. B. Pembe, "Managing pre-eclampsia and eclampsia in Dar es Salaam public health facilities: A focus on equipment, supplies, drugs and knowledge of healthcare workers," *Tanzania Med. J.*, vol. 27, no. 1, pp. 43–54, 2015.

Response to reviewer's comments

Title: Auditing the Readiness of Healthcare Facilities in Antenatal Care Referral and Management of Pre-Eclampsia in Zanzibar- A Protocol for Cross Sectional Study

NB: Line number referenced here are based on the document with track changes

**Comments**	**Response**
**1. Introduction section**	
i. Please focus this part on the facts according to title of you study. Don't repeat the WHO guidelines and available treatment options. Explain what the deficiencies or berries in implementations of these guidelines in your area or hospitals are and how you can rectify them.	This section has been re written line 69-170
ii. Line 90-92, please insert the year to which this data belongs, page 3.	The year is inserted (line 108-110)
iii. Please add reference to the statement made in line 149-150, page 5.	The reference is added (line 164)
**2. Study area section**	
i. Line 208-215, page 8; This statement is repetition of last paragraph of introduction part. So, it can be omitted.	The paragraph is omitted
**3. Variable definition sections**	
i. Please don't repeat the definition of pre-eclampsia (line 262-264, and line 272-276 at page 10. This section needs to be worked on and rephrased.	The section has been omitted but the important information is included in variables measurement section (line 287-305)
ii. Authors still have not made it clear how information will be collected in tool, whether it will be a smartphone or computer-based form or a printed questionnaire will be kept at the hospitals	This information is included in line 346-349
iii. Similarly, there will be different categories of health care workers like medical graduates, nursing staff and other paramedical staff at each hospital. Authors should explain how they will prevent bias while collecting information from them.	For healthcare providers, self-administered questionnaire will be used. The same standardized questionnaire will be used to all categories and results will be presented for each healthcare category. This information is added in line 316-317.
**4. Independent variables**	
i. This section as whole need to be rewritten, sentences is framed very badly. e.g line 298. Page 11; The tool will be adopted from [29] and will be little modified to suit the current study.	The section is re-written line 311-336
**Other comments**	

i. Authors are requested to work on English language in manuscript. Although, they have improved a lot, but much more needs to be done.	The manuscript was sent to English editor
Please use uniform method of writing pre-eclampsi/eclampsia instead of writing PE/E or full form randomly. Similarly use blood pressure instead of writing Blood Pressure.	This has been corrected in the whole document
ii. Authors have not improved reference section. Some references are incomplete. e.g. Reference number at 4, 6, 7, 9, 18, 21, and 25. Please rewrite them.	References are now corrected (number 4,12,13,15,19,23). After editing the text one reference was dropped
