# Supplementary material for: Auditing the readiness of healthcare facilities for referral and management of pre-eclampsia cases in Zanzibar- a study protocol
Source: PLoS One. 2023 Jun 2;18(6):e0286498. doi: 10.1371/journal.pone.0286498 (PMC10237472; doi:10.1371/journal.pone.0286498)
Supplement: Attachment — Submitted filename: point to point response protocol 3.docx [file pone.0286498.s006.docx]

# **Response to reviewer’s comments**

# **Title: Auditing the Readiness of Healthcare Facilities in Antenatal Care Referral and Management of Pre-Eclampsia in Zanzibar- A Protocol for Cross Sectional Study**

*NB: Line number referenced here are based on the document with track changes*

| **Comments** | **Response** |
| --- | --- |
| **1. Introduction section** |  |
| i. Please focus this part on the facts according to title of you study. Don’t repeat the WHO guidelines and available treatment options. Explain what the deficiencies or berries in implementations of these guidelines in your area or hospitals are and how you can rectify them. | This section has been re written line 69-170 |
| ii. Line 90-92, please insert the year to which this data belongs, page 3. | The year is inserted (line 108-110) |
| iii. Please add reference to the statement made in line 149-150, page 5. | The reference is added (line 164) |
| **2. Study area section** |  |
| i. Line 208-215, page 8; This statement is repetition of last paragraph of introduction part. So, it can be omitted. | The paragraph is omitted |
| **3. Variable definition sections** |  |
| i. Please don’t repeat the definition of pre-eclampsia (line 262-264, and line 272-276 at page 10. This section needs to be worked on and rephrased. | The section has been omitted but the important information is included in variables measurement section (line 287-305) |
| ii. Authors still have not made it clear how information will be collected in tool, whether it will be a smartphone or computer-based form or a printed questionnaire will be kept at the hospitals | This information is included in line 346-349 |
| iii. Similarly, there will be different categories of health care workers like medical graduates, nursing staff and other paramedical staff at each hospital. Authors should explain how they will prevent bias while collecting information from them. | For healthcare providers, self-administered questionnaire will be used. The same standardized questionnaire will be used to all categories and results will be presented for each healthcare category. This information is added in line 316-317. |
| **4. Independent variables** |  |
| i. This section as whole need to be rewritten, sentences is framed very badly. e.g line 298. Page 11; The tool will be adopted from [29] and will be little modified to suit the current study. | The section is re-written line 311-336 |
| **Other comments** |  |
| i. Authors are requested to work on English language in manuscript. Although, they have improved a lot, but much more needs to be done. | The manuscript was sent to English editor |
| Please use uniform method of writing pre-eclampsi/eclampsia instead of writing PE/E or full form randomly. Similarly use blood pressure instead of writing Blood Pressure. | This has been corrected in the whole document |
| ii. Authors have not improved reference section. Some references are incomplete. e.g. Reference number at 4, 6, 7, 9, 18, 21, and 25. Please rewrite them. | References are now corrected (number 4,12,13,15,19,23). After editing the text one reference was dropped |
