# Supplementary material for: Auditing the readiness of healthcare facilities for referral and management of pre-eclampsia cases in Zanzibar- a study protocol
Source: PLoS One. 2023 Jun 2;18(6):e0286498. doi: 10.1371/journal.pone.0286498 (PMC10237472; doi:10.1371/journal.pone.0286498)
Supplement: Attachment — Submitted filename: protocol clean third.docx [file pone.0286498.s007.docx]

**Auditing the Readiness of Healthcare Facilities FOR ~~in Antenatal Care~~ Referral and Management of Pre-Eclampsia CASES in Zanzibar- A Protocol ~~for Cross Sectional Study~~**

**TITLE NEEDS REVISION**

Salma A. Rashid^1¶*^, Saada A. Seif^2¶^

^1^ Department of Clinical Nursing, The University of Dodoma, Dodoma, Tanzania

^2^Department of Nursing Management and Education, The University of Dodoma, Dodoma, Tanzania

Funding: No fund received for this study

Competing interest: There is no competing interest to declare

Data availability: Deidentified research data will be made publicly available when the study is completed and published

^*^Corresponding author

Email: sallyrasheed.sr@gmail.com

^¶^These authors contributed equally to this work

**Abstract**

Hypertensive disorders of pregnancy GRAMMAR ??/ MISSING WORD?? reported as the second leading root of maternal morbidity and mortality in Zanzibar. Evidences show that majority of pregnant women in Zanzibar are referred FROM ???? late from antenatal clinics and **most end up**?/with eclampsia SYNTAX / TECHNICAL PROBLEM ??. The **objective NOT OK** of this study is to determine the prevalence of pre-eclampsia WHERE?// , and to assess the readiness of health care facilities **WHERE ???** in relation to pre-eclampsia referral and management in Zanzibar.

This is a descriptive cross-sectional study that will involve a total of 54 healthcare facilities, 176 health care providers and 138 pregnant women with gestation age of 20 weeks and above. Health care facilities will be selected by systematic random sampling, and simple random sampling will be used to select health care providers and pregnant women. Protein in urine test and measurement of blood pressure will be conducted for the diagnosis of pre-eclampsia. Healthcare facilities will be assessed if they meet the requirement for referral and for the availability of equipment and supplies, medications and lab-tests related to the management of pre-eclampsia using a **pre-prepared** ??/ checklist. Questionnaire will be used to determine the proportion of healthcare providers with knowledge and skill on the management of pre-eclampsia within each facility, and to obtain background characteristics information from pregnant women, while patients case files in each facility will be reviewed to assess the management of pre-eclampsia using a pre-prepared checklist **TOO LONG A SENTENCE**??. Data will be analysis using SPSS software version 25. Descriptive statistics will be used to describe the frequency distribution of the study variables and results will be presented in frequency and percentage. Chi square test will be used to describe the relationship between variables and the p-value of < 0.05 will be regarded as statistically significant difference.

**Keywords:**

Pre-eclampsia, eclampsia, healthcare facilities readiness, antenatal care referral, management of pre-eclampsia, Zanzibar.

**Introduction**

Pre-eclampsia is defined as a pregnancy complication characterized by high blood pressure of at least 140mmHg systolic and 90mmHg diastolic pressure measured on two occasions, 4 - 6 hours apart, accompanied by proteinuria of at least 300 mg per 24 hours, or at least +1mg on dipstick tested after 20 weeks of gestation age, and or with swelling of lower limbs, severe headache and or upper abdominal pain and or blurred vision or light sensitivity. While eclampsia is the existence of a new-onset of grand mal-seizure in women with pre-eclampsia [1]. The pathogenesis of pre-eclampsia is not known, though it is associated with abnormal placentation at the beginning of pregnancy, while hypertension and proteinuria occurred ???? **SYNTAX??/** in the second half of pregnancy [2]. The risk factors like obesity, chronic hypertension, diabetics, nulliparous, and twinning pregnancy have been shown to increase the likelihood of developing pre-eclampsia to pregnant women [3].

Pre-eclampsia is categorized as mild and severe, the mild one is diagnosed when blood pressure is greater than 140 systolic or 90 diastolic and 0.3mg of protein is collected in a 24-hour urine sample, or persistent of +1mg protein measurement in urine dipstick. While severe one is diagnosed when the blood pressure is greater than 160/110 mmHg (especially diastolic ≥110 mmHg), proteinuria of 3+mg or greater or 1mg or greater in 24hours, with severe headache, epigastric pain, blurring of vision and vomiting[4]. The prognosis from mild to severe can be fast and unpredictable if left untreated. **REDUNDANT PARAs…DELETE …COME DIRECTLY TO THE POINT**

The prevalence of pre-eclampsia/eclampsia is reported to be much higher in developing countries which ranges from 1.8 to 16.7% [5], while in developed countries like United States of America, the pre-eclampsia prevalence ranges from 3-6% [6]. In East African countries, the prevalence of pre-eclampsia ranges from 6.1% in Kenya [7] to 12.2% in Ethiopia [8]. The recent study done in Tanzania showed the prevalence of severe pre-eclampsia is 4.2% [9] while in Zanzibar the prevalence of pre-eclampsia reported in the year 2011 was 9% [10]. Therefore, there is still need to determine the current prevalence of pre-eclampsia in the country to understand its trend.

Researches show that pre-eclampsia/eclampsia is among the primary cause of maternal and neonatal morbidity and mortality. It is reported to affect 2–8% of pregnancies globally, and it is associated with 10–15% of direct maternal deaths and up to 25% of stillbirths and newborn deaths in developing countries [11], [12]. Tanzania is reported to have 556 maternal deaths per 100,000 live birth [13], and eclampsia related complications accounts for 18.9% to maternal deaths [14]. In Zanzibar, the report of 2008 showed that maternal mortality was 155 deaths per 100,000 live birth [15], while the report of 2017 showed that severe pre-eclampsia was found to contribute to 25.8% of maternal morbidity and 21.8% of maternal mortality [16].

Effective management of pre-eclampsia/eclampsia can be possible by following the WHO guideline of Antenatal Care (ANC) screening for early detection of signs of pre-eclampsia as well as **the** following **the** guideline for pharmacological management and meet the specified **requirement for referral. TOO LONG A SENTENCE??. Poor adherence to these guidelines** can result in poor management of pregnant women with signs of pre-eclampsia and thus may contribute**s** to increasing of maternal morbidity and mortality related to pre-eclampsia/eclampsia. **Grammar?///**

Various factors that affect the management of pre-eclampsia/eclampsia have been identified, **and** these include unavailability of medication **and** supply **and** lack of healthcare providers with adequate knowledge **and** skills for the management of pre-eclampsia. **4 ANDs ikn a sentence!!!!!!**

All these may result in failure to **early detect**, diagnose and providing proper treatment of pre-eclampsia [17], [18]. **GRAMMAR ISSUES**

**NO PROPER LINK / FLOW OF THE THOUGHTS in PARAs**

**Therefore?????,** assessing these factors in each healthcare facility levels is very important step for enhancing good decision making for the distribution of medical supplies ??? related to the management of pre-eclampsia/eclampsia at different healthcare facility levels.

The Ministry of Health Zanzibar adopted the WHO guideline and developed the ‘Management Protocols of Obstetric and Newborn Care’ which is to be uniformly followed at all healthcare facility levels. In addition, health care providers are provided with training on focused antenatal care which include the use of these guidelines. According to this protocol all pregnant women attending ANC must be screened for pre-eclampsia/eclampsia, and those showing signs of pre-eclampsia/eclampsia, must be appropriately managed, and those presented with severe pre-eclampsia at lower healthcare facility levels, must be referred to the next level immediately [19]. However, evidence showed that majority of pregnant women in Zanzibar are referred late and most ends up with eclampsia (25.8%)[16].

**Hence the questions come of**?/// whether all levels of healthcare facilities in Zanzibar are prepared enough in terms of availability of the required equipment, medications, adherence to management and referral guidelines as well as the availability of healthcare providers with knowledge and skills for the management of pre-eclampsia/eclampsia.

**On this ground**, this research **therefore** intends to assess the prevalence of pre-eclampsia and the healthcare facilities readiness in relation to ANC referral and management of pre-eclampsia in Zanzibar. **objective NOT OK**

**Study model: Donabedian model for measuring quality of care**

This study will be guided by model for measuring quality care by Donabedian (Fig. 1) [20]. This model has three approaches to evaluation of the quality of care, which are structure, process, and outcome. The structure which is also known as input measure entails the characteristics of the healthcare facility such as staff to patient ratio and operating times and hours of the services. Process measures reﬂect the way systems work to deliver the desired outcome, and the outcome measure reﬂects the impact on the patient and demonstrates the result of improvement and whether it has ultimately achieved the aim(s) set [20].

The current study will adopt all approaches as specified by the model as follows: The structure measures will be the availability of resources in the health care facilities like skilled and knowledgeable health care providers, availability of guidelines, availability of required tests, resources and equipment, on job training, availability of ambulance, and availability of medication. **The process measure** will be the **adherence to the guideline on the treatment of pre-eclampsia/eclampsia and adherence of guidelines in making the referral** to women with signs of severe pre-eclampsia. **The outcome measure** no link with PROCESS measures listed?????????????????????????!!!!!!!!!!!!!!!!!!!will be the **number of emergency admissions due to severe pre-eclampsia and the number of patients with eclampsia.**

**Methods**

**Study objectives – why repeat here ????**

~~The general objective of this study is to determine the proportion of pre-eclampsia and to assess the readiness of healthcare facilities in relation to pre-eclampsia antenatal care referral and its management in Zanzibar.~~

The Specific objectives are:

1. To determine the prevalence of pre-eclampsia among pregnant women attending ANC. **How can we comment on referral appropriateness …WHEN … prevalence IS BEING ASSESSSED at higher level??? = CONFUSED APPROACH**
2. To assess the availability of equipment, medication and tests related to the management of pre-eclampsia **among all** ??/ healthcare facility levels.
3. To determine proportion of health care providers **working in ANC** ??/with adequate knowledge on the management of pre-eclampsia
4. To determine proportion of health care providers working in ANC with adequate skills on the management of pre-eclampsia
5. **To assess the** QUALITY ?????/ OF **management**?/// of pre-eclampsia in all healthcare facility levels
6. To assess the **status of COMPLIANCE WITH referral standards** among healthcare facilities of lower levels

**Study design**

A descriptive cross-sectional design using a quantitative approach will be used.

**Study area**

This study will be conducted in Zanzibar which is united with and a part of Tanzania. The study setting will be healthcare facilities of all levels in Zanzibar. The healthcare delivery system in Zanzibar is organized into three levels which are the primary level, secondary and tertiary level. The primary level is the lowest level which includes Primary Health Care Unit (PHCU), Primary Health Care Unit Plus (PHCU +) and Primary Health Care Center or Cottage (PHCC). In total, there are 122 PHCU, 34 PHCU+, and 4 PHCC. Primary health care units provide primary health care services and all ANC services including screening of pre-eclampsia, while the PHCU+ provides additional services such as delivery, dental, laboratory, and pharmaceutical services while the PHCCs provide the same services as PHCU+ with the addition of inpatient and X-ray services.

The secondary level which is the middle level of health care delivery includes district hospitals and the regional hospitals. There are two district hospitals and one regional hospital. District hospitals provide second-line referral services including basic surgery, and regional hospitals provide referral points for all district hospitals. The highest level of health care delivery is the tertiary level or called the referral hospital. There is only one referral hospital in Zanzibar which is Mnazi Mmoja National Hospital which provides referral services from the district and regional hospitals.

**Study Population**

This study will involve three types of study populations**.**

1. All pregnant women attending ANC with gestation age of 20 weeks and above
2. All healthcare providers working in ANC units
3. All healthcare facilities of primary, secondary and tertiary level

**Sample size estimation**

**Sample size estimation for pregnant women**

The sample size of pregnant **??????** will be calculated by using formula of Cochran [21]. The proportion of pre-eclampsia of 9%??? **WHERE??** [10], the margin of error of 5% and a Z-score value of 1.96 will be used. Therefore, the sample size will be 138 pregnant women.??????

**Sample size estimation for the health care providers**

The sample size of the health care providers will be calculated by using the **formula Cochran**??// [21]. The proportion of health care providers with **?///** knowledge on the management of pre-eclampsia of 11.8% [22], Z-score value of 1.96 and margin error of 5% will be used. Adding 10% of none response, the minimum sample size of 176 healthcare providers will be used.

**Sample size estimation of healthcare facilities**

The sample size will be calculated using a formula put forward by WHO which states that in assessing lower health facility levels, if there are 100 or fewer, you should study all of them. If there are more than 100 then take at least 30% [23]. In this regard, the total number of primary healthcare facilities in Zanzibar (PHCUs and PHCU+) is 154, therefore only 30% will be taken which is equal to 46 primary healthcare facilities. Moreover, the total number of PHCC is 4, district hospital is 2, the regional hospital is 1, and referral hospital is 1, therefore all of them will be included in the study. Therefore, the total healthcare facilities that will be included in this study will be 54.

**Sampling procedure**

**Selection of pregnant women**

Number of pregnant women per each selected healthcare facility level will be obtained using a proportionate sampling using a formula of Ni = (Ni/Nt)* n [24], whereby Ni = number of pregnant women needed in each facility, Ni = total number of pregnant women attending ANC in each selected facility per month, Nt = the total number of pregnant women attending ANC in all selected facilities per one month and n = the estimated sample size of pregnant women. Within healthcare facility, simple random sampling will be used to select study participants.

**Selection of health care providers**

Number of health care providers per each selected healthcare facility and from each professional cadre will be obtained using a proportionate sampling [24] whereby Ni = number of health care providers needed from each facility and from each cadre, Ni = total number of health care providers working in ANC in each cadre from each healthcare facility, Nt = total number of health care providers working in ANC in all selected facilities for each cadre, and n= the estimated sample size. Within the healthcare facility, all healthcare providers who met the inclusion criteria will be selected using simple random sampling.

**Selection of healthcare facilities**

All health centers (PHCCs), district hospitals, regional hospital and referral hospital will be included in this study. Moreover, systematic random sampling method will be used to select PHCUs whereby the sampling frame will be a total number of PHCUs and the k^th^ interval will be calculated using the formula of K^th.^ = N/n [25].

**Measurement of Variables**

**Dependent Variables ?//**

**Pre-eclampsia:** This will be measured by the blood pressure reading of at least 140mmHg systolic and 90mmHg diastolic, measured on two occasions; **4 - 6 hours apart**,**WILL THEY BE AVAILBLE FOR SO LONG IN ANC?/**/ accompanied by proteinuria of at least +1mg on dipstick, and or severe headache or upper abdominal pain or blurred vision or light sensitivity. Nominal dichotomous scale will be used and the findings will be categorized into positive and negative for pre-eclampsia.

**Management of pre-eclampsia:** This refers to all treatment, measurements and tests done to a pregnant woman with signs of pre-eclampsia as per the MOHWZ guideline. This variable will be measured by 16 YES/NO items in **ratio scale**??// assessing whether management of pre-eclampsia was conducted **as per the established guideline?? 1,2,3 OR 7 / 16????**, which includes provision of proper medications, monitoring of blood pressure and fetal heart rate, checking protein in urine and conducting liver and kidney function tests [4]. Five items will be for mild pre-eclampsia and eleven items for severe pre-eclampsia. One point will be awarded for the correct act carried out and 0 for the incorrect or missed acts. For mild pre-eclampsia a total score of **5 points will be regarded as proper management** = **GIVE REFERENCE** , and for severe pre-eclampsia a total score of 11 points will be regarded as proper management, less than that is improper management.

**Status of meeting referral requirement:** In this study, **it is when** ?//the healthcare facility meets all required referral standards to refer a woman with signs of pre-eclampsia/eclampsia based on the recommended guidelines. This will be measured by 8 YES/NO items in nominal scale assessing whether the referrals requirements are met as per the established guideline. The tool will be adopted from WHO, (2013), and it includes assessing if the facility has an established clear referral pathway, provide pre-referral treatment to the patient, refer the patient accompanied by health care provider, has referral guideline and has ambulance.

**Independent Variables**

**Knowledge of pre-eclampsia:** This will be measured by 10 multiple choice questions in ratio scale. The standardized structured questionnaire will be adopted from Olaoye et al. [26] which has a Cronbach alpha was 0.72. One point will be awarded for the correct answer and zero point for the incorrect answer. Example of the question is ‘What is the loading dose of magnesium sulfate?’ The total score will be 10, and 75^th^ percentile will be used to categories into those with adequate knowledge and inadequate knowledge. The same questionnaire will used to all categories of healthcare providers and the knowledge level will be presented for each category.

**Skills for management of pre-eclampsia:** This will be measured by 7 items in the form of task questions. The tool will be adopted from Jhpiego, 2017 [27]. Participants will be required to describe in details all the important steps in carrying out a certain procedure related to the management of pre-eclampsia. Example of the question is ‘What are the steps for providing the loading dose of magnesium sulphate?’ One point will be awarded for the complete mentioned steps and zero point for incomplete, incorrect or missed step. The total score will be 7 and 75^th^ percentile will be used to categorize those with adequate skills against those with inadequate skills.

**Availability of Equipment, tests and medication:** These will be measured by 27 items on dichotomous nominal scale on the availability of well-functioning equipment necessary for the management of pre-eclampsia (14 items, e.g. working blood pressure machine, working fetoscope etc), the availability of essential medications (10 items, e.g. methyldopa and diazepam) and the availability of tests needed for the management of pre-eclampsia (3 items, eg. protein in urine test and kidney function test). The tool will be adopted from Maembe and Pembe, 2015 [28].

**Background characteristics:** The background characteristics of healthcare providers will be measured by variables from literature which will include years of working experience in ANC, working healthcare facility level, and ever attended training on the management of pre-eclampsia. The background characteristics of pregnant women will include number of ANC visits, having a history of pre-eclampsia, and having a history of using medication in the current pregnancy.

**Data collection procedure:**

Data will be collected within the selected healthcare facilities for the period of four weeks. The principal investigator and six research assistants who are registered nurses will collect the data. In this study, several methods of data collection methods will be used depending on the type of data needed to answer a particular study objective as follows:

**Questionnaire:** Self-administered questionnaire will be used to obtain data from healthcare providers for assessing their knowledge and skills on the management of pre-eclampsia while interviewer-administered questionnaire will be used to obtain data from pregnant women for assessing their background characteristics and signs and symptoms related to hypertensive disorders of pregnancy. Printed questionnaire for healthcare providers will be distributed in each healthcare facility and for pregnant women research assistant will be reading the questions and write down the response from participants.

Measurement method: This method will be used to get data on blood pressure readings of pregnant women to be used for making diagnosis of pre-eclampsia.

**Is this team different from the treating team??/**

**Will they be involved in management of the cases? If yes, it will bias the results!!**

In this method a research assistant will instruct a pregnant woman to relax at least ten minutes and sit comfortable in an up-right position with her back and arm supported and both legs flat on the floor. Moreover, a woman will be instructed to remove any tight clothes from around her arm. The appropriate cuff size will be used where by cuff should encircle at least three-fourths of the circumference of arm in a 2 cm above elbow. The measurement will be taken by using digital blood pressure machine and will be taken twice at least four hours apart.

**Chemical investigations method:** Urinalysis with visual reagent strip, “dipstick” will be used to collect data on protein in urine. This data will be used together with other information to make diagnosis of pre-eclampsia. In this method a pregnant woman will be given a urine bottle and will be instructed on how to collect a clean-catch urine sample. The research assistant will dip the end of the dipstick into the urine sample and shake off any excess urine by tapping the dipstick on the side of the urine bottle. After thirty seconds a comparison of dipstick pad with the color chart on the dipstick container will be done to check for the presence of protein in urine.

**Documentary review:** This method will be used to obtain information on the management of pre-eclampsia whereby the principle investigator/research assistant will review the case files of patients **diagnosed with pre-eclampsia BY ?///**, should it be mild or severe pre-eclampsia to look for management provided against the standard treatment guideline using pre-prepared checklist.

**Observation method:** This method will be used for assessing the availability of equipment, tests and medications related to the management of pre-eclampsia using a pre-prepared checklist. The same method will be used to assess whether the facility meets the referral standards. In this method, the principal investigator/research assistant will inspect the facility and record the available equipment, medication, tests, guidelines and referral practice records the pre-prepared checklists. **HAWTHORNE EFFECT ???**

**Data management plan**

Data obtained from participants will be secured in safe computer with password that will be able to be accessed by the researcher’s team only and all safety data protocols of the University of Dodoma will be taking under consideration in order to secure participants information.

**Data analysis plan**

Data will be analyzed using SPSS software version 25. Data will be cleaned and checked for completeness before subjected to models of analysis. Descriptive statistics will be used to describe the proportion of pre-eclampsia, the frequency distribution of equipment, medication, tests, referral standards, and knowledge and skills of health care providers on the management of pre-eclampsia. Chi-square test on the other hand will be used to show the **association between pre-eclampsia and demographic characteristics of pregnant women.** LINK WITH THE OBJECTIVES – NO SUCH PLAN is there???

The same test will be used to show association between health care facility levels and management of pre-eclampsia, availability of equipment, test and medication, and availability of skilled and knowledgeable health care providers. In addition, chi square test will be used to show the association between knowledge and skills of health care providers on the management of pre-eclampsia and their background characteristics. In order to avoid hospital bias, the data collector will not be among nurses working in the selected facility, confidentiality from respondents will be assured and data will be analyzed by considering the level of healthcare facility, that is the findings for each healthcare facility level will be presented separately.

**Ethical consideration and declaration**

Ethical permission for this study was obtained from the University of Dodoma Research Ethics Committee (UDOM-REC) with reference number MA.84/261/02/218 and Zanzibar health research institute (ZAHRI) with a reference number ZAHREC/04/ST/JAN/2021/02. A written consent will be given to health care providers and pregnant women after explaining the purpose of the study and being told that their participation is voluntary and they can be able to withdrawal any time from the study.

**Status and timeline of the study**

The study is expected to be conducted for the period of four weeks, on the day of submission the status was in planning for data collection.

**~~Discussion~~**

**Strength and limitation**

The strength of this study is that it will involve all healthcare facilities in Zanzibar, form lower level to the highest level. This is important because the results that will be obtained will portray the true picture of the current status of pre-eclampsia management in healthcare facilities in Zanzibar. To the best of the authors knowledge, this will be the first study to be conducted in Zanzibar for auditing healthcare facilities in relation to pre-eclampsia management and referral. This information will help the Ministry of Health-Zanzibar to make proper decisions to strengthen healthcare services to pregnant women in terms of increasing the number of skilled health care providers, medication, equipment and tests. All these will contribute to minimize the delay of receiving appropriate management hence decreasing the burden of pre-eclampsia/eclampsia complication. The limitation of this study design will be the difficulty to tally which patient who developed eclampsia was managed by which healthcare provider, therefore we will not be able to tell whether patient’s complication was associated with poor service provided by unskilled healthcare provider or it is because of women failure to adhere to the management provided.

**Dissemination plan**

Finding obtained from this study will be disseminated to the University of Dodoma, to the Ministry of health Zanzibar and will be submitted for publishing in peer reviewed journal.

**How amendments to the study, including termination, will be dealt with**

If any amendments should be made to the study including termination of the study, this will be communicated to the journal auditoria and submit the changes or give reason for the termination.

**Author’s contributions**

S.A.R designed the study and wrote the protocol for ethical approval. S.A.S reviewed and provide constructive inputs to improve this protocol for publication.

**Acknowledgements**

We acknowledge the support of Tasakhtaa Global Hospital-Zanzibar for the financial support to undertake master degree which lead to the production of this article.

**Supporting information**

Questionnaire for assessing knowledge and skills of healthcare providers

Questionnaire for assessing background characteristics of pregnant women

Checklist for assessing healthcare facilities for availability of medication, equipment and test

Checklist for assessing management of pre-eclampsia

Checklist for assessing the referral standard

**References:**

[1]	ACOG Committee on Obstetric Practice, “Practice bulletin #33: diagnosis and management of preeclampsia and eclampsia,” *Obstet. Gynecol.*, vol. 99, no. 1, pp. 159–167, 2002, doi: 10.1016/s0029-7844(01)01747-1.

[2]	J. M. Roberts, “Contemporary Concepts of the Pathogenesis and Management of Preeclampsia,” no. June, pp. 3183–3186, 2014, doi: 10.1001/jama.287.24.3183.

[3]	V. L. Bilano, E. Ota, T. Ganchimeg, R. Mori, and J. P. Souza, “Risk factors of pre-eclampsia/eclampsia and its adverse outcomes in low- and middle-income countries: A WHO secondary analysis,” *PLoS One*, vol. 9, no. 3, pp. 1–9, 2014, doi: 10.1371/journal.pone.0091198.

[4]	MHCDGEC, "Standard treatment guidelines & national essential medicines list, Tanzania mainland, 453. 2019, Retrieved from <https://apps.who.int/medicinedocs/documents/s23541en/s23541en.pdf> [5]	L. Say et al., “Global causes of maternal death: A WHO systematic analysis,” Lancet Glob. Heal., vol. 2, no. 6, pp. 323–333, 2014, doi: 10.1016/S2214-109X(14)70227-X. [5]	J. Wandabwa, P. Doyle, P. Kiondo, O. Campbell, N. Maconichie, and G. Welishe, “Risk factors for severe pre-eclampsia and eclampsia in Mulago Hospital, Kampala, Uganda.,” *East Afr. Med. J.*, vol. 87, no. 10, pp. 415–424, 2010, doi: 10.4314/eamj.v87i10.

[6]	K. R. Fingar, I. Mabry-Hernandez, Q. Ngo-Metzger, T. Wolff, C. A. Steiner, and A. Elixhauser, “Delivery Hospitalizations Involving Preeclampsia and Eclampsia, 2005–2014: Statistical Brief #222,” *Healthc. Cost Util. Proj. Stat. Briefs*, pp. 1–26, 2006.

[7]	A. N. Otieno, “Factors Contibuting To Adverse Outcomes Of Pre-Eclampsia Among Pregnant Women Attending Antenatal Clinics In Kibera Slums, Nairobi,” 2012.

[8]	A. S. Belay and T. Wudad, “Prevalence and associated factors of pre- eclampsia among pregnant women attending anti-natal care at Mettu Karl referal hospital , Ethiopia : cross-sectional study,” pp. 1–8, 2019.

[9]	I. O. Frank, J. M. Michael, R. Jenny, and O. Joseph, “Prevalence and risk factors for pre-eclampsia/eclampsia in Northern Tanzania,” *J. Public Heal. Epidemiol.*, vol. 12, no. 2, pp. 78–85, 2020, doi: 10.5897/jphe2019.1143.

[10]	N. Tufton and R. R. Patel, “Prevalence of hypertensive disorders in a prenatal clinic in Zanzibar,” *Int. J. Gynecol. Obstet.*, vol. 112, no. 1, pp. 69–70, 2011, doi: 10.1016/j.ijgo.2010.09.005.

[11]	L. Say *et al.*, “Global causes of maternal death: A WHO systematic analysis,” *Lancet Glob. Heal.*, vol. 2, no. 6, pp. 323–333, 2014, doi: 10.1016/S2214-109X(14)70227-X.

[12]	World Health Organization, "Trends in Maternal Mortality 2000 to 2017, pp. 16, 2019, [Online] Retrieved from <https://apps.who.int/iris/bitstream/handle/10665/327596/WHO-RHR-19.23-eng.pdf>

[13]	MHCDGEC “Tanzania Demographic and Health Survey and Malaria Indicator Survey 2015-2016 - Final Report [FR321] | Enhanced Reader.” Retrieved from moz-extension://a6710f23-f161-476e-ae79-432076c7f649/enhanced-reader.html?openApp&pdf=https%3A%2F%2Fdhsprogram.com%2Fpubs%2Fpdf%2FFR321%2FFR321.pdf [14]	D. A. M. Makuwani *et al.*, “Baseline Data on Trend of Maternal Mortality in Tanzania using Administrative Data and its Policy Implication. 2018 Report,” *Glob. J. Med. Res.*, vol. 20, no. 6, pp. 5–12, 2020, doi: 10.34257/gjmrkvol20is6pg5.

[15]	MoHSW, "Zanzibar Health Management Information System Unit; Health Information Bulletin 2008", 2009, Retrieved from <https://mohz.go.tz/eng/health-bulletins/> [16]	T. Herklots, L. Van Acht, T. Meguid, A. Franx, and B. Jacod, “Severe maternal morbidity in Zanzibar ’ s referral hospital : Measuring the impact of in- hospital care,” pp. 1–11, 2017.

[17]	A. Barua, S. Mundle, H. Bracken, T. Easterling, and B. Winikoff, “Facility and personnel factors influencing magnesium sulfate use for eclampsia and pre-eclampsia in 3 Indian hospitals,” *Int. J. Gynecol. Obstet.*, vol. 115, no. 3, pp. 231–234, 2011, doi: 10.1016/j.ijgo.2011.07.016.

[18]	O. Oguntunde *et al.*, “Factors influencing the use of magnesium sulphate in pre-eclampsia/eclampsia management in health facilities in Northern Nigeria: A mixed methods study,” *BMC Pregnancy Childbirth*, vol. 15, no. 1, pp. 1–8, 2015, doi: 10.1186/s12884-015-0554-8.

[19]	Goverment of Zanzibar, “Management protocols for emergency obstetric and newborn care,” Zanzibar, 2019. Retrieved from <https://mohz.go.tz/wp-content/uploads/2021/01/Zanzibar-Reproductive-Maternal-Newborn.pdf> [20]	NHSI (NHS Improvement) ACT Academy, “Quality, Service Improvement and Redesign Tools: A model for measuring quality care,” *Nhsi Act*, pp. 198–201, 2008.

[21]	W. G. Cochran, “Double sampling,” *Cochran WG. Sampl. Tech. 3rd ed. New York John Wiley Sons, Inc*, pp. 327–358, 1977.

[22]	S. Liljevik and E. S. V. B. Lohre, “Evaluation of knowledge and management practices of hypertension in pregnancy among health care workers in Moshi urban, Tanzania.,” pp. 1–41, 2012.

[23]	WHO, "Monitoring Emergency Obstetric Care: a Handbook", 2009, Retrieved from http://whqlibdoc.who.int/publications/2009/9789241547734_eng.pdf

[24]	R. Pandey and M. R. Verma, “Samples allocation in different strata for impact evaluation of developmental programme,” *Rev. Mat. Estat*, vol. 26, no. 4, pp. 103–112, 2008.

[25]	R. Iachan, “Systematic Sampling: A Critical Review,” *Int. Stat. Rev. / Rev. Int. Stat.*, vol. 50, no. 3, p. 293, Dec. 1982, doi: 10.2307/1402499.

[26]	T. Olaoye, O. O. Oyerinde, O. J. Elebuji, and O. Ologun, “Knowledge, Perception and Management of Pre-eclampsia among Health Care Providers in a Maternity Hospital,” *Int. J. MCH AIDS*, vol. 8, no. 2, pp. 80–88, 2019, doi: 10.21106/ijma.275.

[27]	“Pre-eclampsia & Eclampsia (HMS PE&E) – Helping Mothers Survive.” https://hms.jhpiego.org/pre-eclampsia_eclampsia/ (accessed Dec. 29, 2021).

[28]	L. E. Maembe and A. B. Pembe, “Managing pre-eclampsia and eclampsia in Dar es Salaam public health facilities: A focus on equipment, supplies, drugs and knowledge of healthcare workers,” *Tanzania Med. J.*, vol. 27, no. 1, pp. 43–54, 2015.
