# Supplementary material for: Auditing the readiness of healthcare facilities for referral and management of pre-eclampsia cases in Zanzibar- a study protocol
Source: PLoS One. 2023 Jun 2;18(6):e0286498. doi: 10.1371/journal.pone.0286498 (PMC10237472; doi:10.1371/journal.pone.0286498)
Supplement: Attachment — Submitted filename: point to point response protocol fourth.docx [file pone.0286498.s009.docx]

# **Response to reviewer’s comments**

# **Title: Auditing the Readiness of Healthcare Facilities for Referral and Management of Pre-Eclampsia Cases in Zanzibar- A study Protocol**

*NB: Line number referenced here are based on the document with track changes*

| **Comments** | **Response** |
| --- | --- |
| **Reviewer 1** |  |
| **1. Introduction section** |  |
| i. Please focus this part on the facts according to title of you study. Don’t repeat the WHO guidelines and available treatment options. Explain what the deficiencies or berries in implementations of these guidelines in your area or hospitals are and how you can rectify them. | This section has been re written line 80-158 |
| ii. Line 90-92, please insert the year to which this data belongs, page 3. | The year is inserted (line 115-118) |
| iii. Please add reference to the statement made in line 149-150, page 5. | The reference is added (line 150) |
| **2. Study area section** |  |
| i. Line 208-215, page 8; This statement is repetition of last paragraph of introduction part. So, it can be omitted. | The paragraph is omitted |
| **3. Variable definition sections** |  |
| i. Please don’t repeat the definition of pre-eclampsia (line 262-264, and line 272-276 at page 10. This section needs to be worked on and rephrased. | The section has been omitted |
| ii. Authors still have not made it clear how information will be collected in tool, whether it will be a smartphone or computer-based form or a printed questionnaire will be kept at the hospitals | This information is included in line 338-339 |
| iii. Similarly, there will be different categories of health care workers like medical graduates, nursing staff and other paramedical staff at each hospital. Authors should explain how they will prevent bias while collecting information from them. | For healthcare providers, self-administered questionnaire will be used. The same standardized questionnaire will be used to all categories and results will be presented for each healthcare category. This information is added in line 306-307. |
| **4. Independent variables** |  |
| i. This section as whole need to be rewritten, sentences is framed very badly. e.g line 298. Page 11; The tool will be adopted from [29] and will be little modified to suit the current study. | The section is re-written line 300-322 |
| **Other comments** |  |
| i. Authors are requested to work on English language in manuscript. Although, they have improved a lot, but much more needs to be done. | The manuscript was sent to English editor |
| Please use uniform method of writing pre-eclampsi/eclampsia instead of writing PE/E or full form randomly. Similarly use blood pressure instead of writing Blood Pressure. | This has been corrected in the whole document |
| ii. Authors have not improved reference section. Some references are incomplete. e.g. Reference number at 4, 6, 7, 9, 18, 21, and 25. Please rewrite them. | References are now corrected (number 11,12,14,18, 19,23,24). After editing the text one reference was dropped |
| **Reviewer 2** |  |
| Title needs revision | The title is revised to read: Auditing the Readiness of Healthcare Facilities for Referral and Management of Pre-Eclampsia Cases sin Zanzibar- A study Protocol |
| ***Comments on Track changes document:*** |  |
| Abstract: grammatical errors | Grammar correction was applied to the entire document. |
| Introduction: |  |
| - paragraph 2 is a redundant | The paragraph is deleted, line 93-99 |
| - grammatical errors | Grammar correction was applied to the entire document. |
| - model used: the outcome measure does not link with the process measure | Correction has been made, only two domains of the model will be used, the structure and the process. The outcome is left out, line 168-175 |
| Objectives:   - need to be corrected | The objective of the prevalence of pre-eclampsia is omitted, line 179-184 |
| Variable measurement   - need revision - give reference for categorizing the management into proper and improper management | - The variable measurement of pre-eclampsia is omitted since the objective was omitted, line 275-279 - We used a score of 100% as proper, less than that is improper, line 289-291. |
| Data collection: the observation method, how will you control the Hawthorne effect? | - The observation method will be used to assess the availability of equipment and supplies and tests; therefore, Hawthorne effect is not expected, line 367-369 |
| **Reviewer 3** |  |
| **Abstract**  • Future tense has to be used at some places. | Future tense is now used, line 49 |
| **Introduction** |  |
| Minor grammatical corrections required | Grammar correction was applied to the entire document. |
| The more recent data from Zanzibar should be quoted | The more available data are now used, line 107-108, 115-116 |
| **Methods** |  |
| The bullets could be replaced by plain text | The bullets are removed, line 179-184 |
| Sample size estimation | Sample size estimation for pregnant women was removed since the objective was omitted line 228-231 |
| The various paragraphs could be clubbed | Paragraph are reduced in number |
| **Analysis of the response** |  |
| The percentile part has to be removed  The threshold has to be defined to label as acceptable. | This statement has been removed, line 305, 314 |
| **References** |  |
| Please correct ref no 4 and 5 | Reference has been deleted because the paragraph was deleted. |
| **Reviewer 4** |  |
| The study topic is interesting especially in resource poor settings. The study protocol needs language editing and along sentence reframing as mentioned by fellow reviewers. Please resubmit with revisions. | Grammar correction was applied to the entire document. |
| **Reviewer 5** |  |
| There are grammatical corrections required, cited some:   - Abstract (bottom up fifth line) simple grammatical correction: Data will be ‘analyzed’ using SPSS software. - Line 105: may ‘contribute’ to - Line 110: Reframe grammatically if possible - Line 351: the results obtained will portray | Grammar correction was applied to the entire document.   - This statement has been corrected, line 70 - The paragraph was deleted, line 127 - The statement has been corrected, line: 132-133 |
