# Supplementary material for: Auditing the readiness of healthcare facilities for referral and management of pre-eclampsia cases in Zanzibar- a study protocol
Source: PLoS One. 2023 Jun 2;18(6):e0286498. doi: 10.1371/journal.pone.0286498 (PMC10237472; doi:10.1371/journal.pone.0286498)
Supplement: Attachment — Submitted filename: RESPONSE TO REVIEWER comments -salma.docx [file pone.0286498.s005.docx]

**RESPONSE TO REVIEWER’S COMMENTS**

We would like to take this opportunity to thank you all, editors and reviewers from PlosOne’ publication team for your efforts devoted to review our protocol. We appreciate for all the comments given. We have thoroughly responded to all reviewers’ comments. The table below is a summary of point by point responses for the reviewers.

| **COMMENTS** | **RESPONSE** |
| --- | --- |
| 1. The document is full of English language grammatical errors, which should be corrected. | The grammatical errors have been corrected |
| 2. Scientific English language need to be improved throughout the paper. | The scientific English language has been improved |
| 3. Please use uniform method of references in paper. | The Vancouver style is now used for the whole document |
| 4. Please elaborate the method sections for following points. | |
| • Please add duration of study. | The duration of the study is added on page 12 line 317 |
| • Please describe who will collect data. | The data collection procedure is now improved to include those who will collect the data on page 12 line 318 |
| • Rewrite the dependent and independent variables, presently this part is written haphazardly. | This part has been re-written and made clear (page 10-11, line 271-315 |
| • Please state the guidelines/definitions explicitly those are being used at your peripheral hospital while managing hypertensive disorder in pregnant women. Also, state whether these guidelines are uniformly followed at all centers or there are some differences in the protocols. | The Ministry of Health Zanzibar developed a ‘Management Protocol for Emergency Obstetric and Newborn Care’ to be followed uniformly in all health centers of lower and high levels. This information is now included in page 5, line 142-144. |
| • Please state whether some training/orientation program exist for the peripheral health workers for providing antenatal care. | Yes there was a training program on ‘Focused Antenatal Care’ that is provided to all health care providers working in ANC. This information is now included in page 5 line 144-145 |
| • Please describe the process to avoid hospital bias. | In order to avoid hospital bias, the following mechanism will be used:   - The data collector will not be among nurses working in the selected facility - Confidentiality from respondents will be assured - Data will be analyzed by considering the level of healthcare facility. That is the findings for each healthcare facility level will be analyzed separately (page 13, line 361-367) |
| • Please describe groups for statistical analysis and how the comparison will be performed. | - Descriptive statistics will be used to describe the proportion of pre-eclampsia, the frequency distribution of equipment, medication, tests, referral standards, and knowledge and skills of health care providers on the management of pre-eclampsia. - Chi-square test will be used to show association between:   - Pre-eclampsia vs. demographic characteristics of pregnant women   - health care facility levels vs. management of pre-eclampsia   - health care facility levels vs. availability of equipment, test and medication   - health care facility levels vs. availability of skilled and knowledgeable health care providers   - knowledge vs demographic characteristics of health care providers   - skills vs. demographic characteristics of health care providers   (page 13, line 353-361) |
